# Deep learning-based segmentation of ultra-low-dose CT images using an optimized nnU-Net model

**DOI:** 10.1007/s11547-025-01989-x

**Published:** 2025-03-18

**Authors:** Yazdan Salimi, Zahra Mansouri, Chang Sun, Amirhossein Sanaat, Mohammadhossein Yazdanpanah, Hossein Shooli, René Nkoulou, Sana Boudabbous, Habib Zaidi

**Affiliations:** 1 Division of Nuclear Medicine and Molecular Imaging, Geneva University Hospital, CH-1211 Geneva, Switzerland; 2 School of Information and Communication Engineering, Beijing University of Posts and Telecommunications, Beijing, 100876 China; 3 Department of Radiology, Shiraz University of Medical Sciences, Shiraz, Iran; 4 Department of Radiology, Bushehr University of Medical Sciences, Bushehr, Iran; 5 Division of Radiology, Geneva University Hospital, CH-1211 Geneva, Switzerland; 6 Department of Nuclear Medicine and Molecular Imaging, University of Groningen, University Medical Center Groningen, Groningen, The Netherlands; 7 Department of Nuclear Medicine, University of Southern Denmark, Odense, Denmark; 8 University Research and Innovation Center, Óbuda University, Budapest, Hungary

**Keywords:** Ultra-low-dose CT, Organ segmentation, Radiation dose, Deep learning, nnU-Net

## Abstract

**Purpose:**

Low-dose CT protocols are widely used for emergency imaging, follow-ups, and attenuation correction in hybrid PET/CT and SPECT/CT imaging. However, low-dose CT images often suffer from reduced quality depending on acquisition and patient attenuation parameters. Deep learning (DL)-based organ segmentation models are typically trained on high-quality images, with limited dedicated models for noisy CT images. This study aimed to develop a DL pipeline for organ segmentation on ultra-low-dose CT images.

**Materials and methods:**

274 CT raw datasets were reconstructed using Siemens ReconCT software with ADMIRE iterative algorithm, generating full-dose (FD-CT) and simulated low-dose (LD-CT) images at 1%, 2%, 5%, and 10% of the original tube current. Existing FD-nnU-Net models segmented 22 organs on FD-CT images, serving as reference masks for training new LD-nnU-Net models using LD-CT images. Three models were trained for bony tissue (6 organs), soft-tissue (15 organs), and body contour segmentation. The segmented masks from LD-CT were compared to FD-CT as standard of reference. External datasets with actual LD-CT images were also segmented and compared.

**Results:**

FD-nnU-Net performance declined with reduced radiation dose, especially below 10% (5 mAs). LD-nnU-Net achieved average Dice scores of 0.937 ± 0.049 (bony tissues), 0.905 ± 0.117 (soft-tissues), and 0.984 ± 0.023 (body contour). LD models outperformed FD models on external datasets.

**Conclusion:**

Conventional FD-nnU-Net models performed poorly on LD-CT images. Dedicated LD-nnU-Net models demonstrated superior performance across cross-validation and external evaluations, enabling accurate segmentation of ultra-low-dose CT images. The trained models are available on our GitHub page.

**Supplementary Information:**

The online version contains supplementary material available at 10.1007/s11547-025-01989-x.

## Introduction

Artificial intelligence (AI) has shown a promising potential in facilitating a variety of tasks in medical imaging, particularly in image segmentation, including both normal and abnormal tissue delineation [1–3]. Organ segmentation has a vital role in quantitative imaging, kinetic modeling and personalized dosimetry in diagnostic and theranostic radiology [4–10]. Numerous recent studies focused on deep learning (DL)-based organ segmentation of CT images, using both local and publicly available datasets [11–14]. Some of these studies reported promising results [2, 15–18] and have been adopted by other research groups for different tasks [19–21]. The nnU-Net [22] pipeline has shown highly robust performance, and most recent studies have employed this model to implement their solutions.

Despite its undeniable diagnostic potential and versatile applications, concerns about the radiation dose persist, as CT is still considered as a high-dose medical imaging modality [23, 24]. Low-dose (LD) CT imaging is commonly used in emergency imaging, on radiation-sensitive groups, such as pediatrics and pregnant patients, in total-body imaging for patient follow-ups, and primarily in hybrid imaging in CT-based attenuation correction (CTAC) on hybrid SPECT/CT and PET/CT scanners [25–28]. In addition, whole-body LD-CT is well established and recommended in international guidelines for multiple myeloma screening based on consensus reports from the International Myeloma Working Group [29]. Lowering the radiation dose can be achieved by optimizing CT acquisition parameters, such as lowering tube potential (kVp), reducing the tube current (mAs), increasing the pitch, and using sparse acquisition techniques [30]. These adjustments change the noise and contrast patterns in CT images, potentially generating artifacts, such as streak and beam hardening artifacts, in the reconstructed CT images. However, ultra-low-dose CTAC proved to be sufficient for attenuation correction in both PET and SPECT imaging [26, 31]. In dynamic PET and SPECT protocols, where emission data acquisition might be prolonged, potentially increasing the likelihood of patient movement, multiple CT scans might be needed for attenuation correction and anatomical localization. Multiple time-point SPECT imaging is common in theranostics, and dynamic 3D reconstruction has become recently possible with the introduction of multi-pinhole SPECT cameras [32]. Most of these repeated CT acquisitions could be acquired at lower radiation doses to address concerns related to potential radiation hazards and radiation carcinogenic effects. Selfridge et al. [33] acquired 5 mAs (tube current × tube rotation time) CT scans for repeated scans in addition to a higher-dose semi-diagnostic scan at one time point. The segmentation masks from the first high-dose CT could be less useful for the following dynamic PET and SPECT acquisitions because of voluntary and involuntary mismatches between the scans. This fact leverages the need for a reliable segmentation model on low-dose and ultra-low-dose images.

Cevora et al. evaluated the performance of deep learning-based organ segmentation models and reported that the performance depended on gender, image characteristics, and dataset origin, including scanner type, acquisition and reconstruction parameters, as well as patient population features [34]. Tsanda et al. [35] reported a minor reduction of 3% of the Dice score on models developed by Wasserthal et al. [17] when applied on LD-CT images where the tube current was reduced to 20% of the original diagnostic contrast enhanced CTs [35]. They simulated diagnostic LD-CT images with almost 80 mAs, which is similar to values reported for routine semi-diagnostic PET/CT and SPECT/CT acquisitions [36]. This shows that available DL-based segmentation models work adequately on semi-diagnostic CT images with minimal drop in performance. However, the effectiveness of available DL organ segmentation models needs to be validated for ultra-low-dose CT images.

To the best of our knowledge there is a deficit of automated segmentation models dedicated for low-dose or ultra-low-dose CT images, and as such, studies evaluating the performance of DL segmentation models on ultra-low-dose CT images are lacking. The usefulness of DL models on non-contrast enhanced low-dose and ultra-low-dose CT images, especially ultra-low-dose CTAC images may be questioned. We recently developed an nnU-Net multiple organ segmentation model using a large training dataset (> 4000 images), including adult and pediatric cases [2]. This study aimed to investigate the performance of these models on ultra-low-dose CT images using realistic CT images and to develop a dedicated nnU-Net pipeline for image segmentation in LD-CT scanning scenarios.

## Methods and materials

### Patients’ population and CT protocols

A total of 274 raw CT scans (projections) from 212 separate patients were collected from three Siemens scanners located at the Nuclear Medicine and Radiology Departments of Geneva University Hospital, Geneva, Switzerland. Siemens ReconCT software is a certified version of Siemens (Siemens AG, Germany) research CT reconstruction tool. This software allows adding scanner-specific noise to raw projections at each kVp, thus generating sinograms corresponding to lower tube current acquisitions. It should be noted that this scanner-specific noise is different from adding Poisson or Gaussian noise and is measured according to phantom calibration results. ReconCT software has a command line interface (CLI) for reconstructing simulated low tube current CT images, from 99 to 1% of the original acquisition tube current. The raw data were reconstructed using Siemens CLI with ADMIRE reconstruction algorithm, 1 mm slice thickness, a 50 cm field-of-view, and 512 × 512-pixel size resulting in 0.97 mm pixel dimension on axial slices, i.e., 0.97 × 0.97 × 1 mm^3^ voxel spacing. CT raw data were reconstructed once with the standard full-dose sinograms (FD-CT) and once with the scanner-specific noise added to sinograms prior to reconstruction corresponding to tube currents of 50%, 30%, 25%, 20%, 15%, 10%, 5%, 4%, 3%, 2%, and 1% of the original acquisition. We denote the different dose levels as LD-CT-X%, where X represents the percentage of dose level reduction. For the dose levels lower than 10%, the ultra-low-dose option as well as electrical noise was activated in the software configuration file; the rest of parameters remain unchanged.

Details about the algorithm used by Siemens ReconCT application are described in Stierstorfer et al. [37].Overall, 21 organs were delineated on reference FD-CT images using pretrained nnU-Net models (FD-nnU-Net) [2]. The organs included the clavicles, femoral heads, hips, sacrum, rib cage, and vertebrae, adrenal glands (AG), aorta, brain, colon, eyeballs, gall bladder (GB), kidneys, liver, lungs, pancreas, erector spinae, small bowel, spleen, stomach, urinary bladder (UB), and heart. The segmentations were co-registered to LD-CT images as the reconstruction parameters and location information were similar. The segmentation masks generated on FD-CT images were considered as reference masks for training new segmentation models. The demographic information of the included patients is summarized in Table 1. Information about the validation of the low-dose CT simulation process is presented in supplementary Tables 1 and 2.

**Table 1:** Demographic information of patients included in this study Recon. Diameter: Reconstruction diameter. Same means that the parameter was kept constant for all dose levels

Parameters	DoseLevel-1%	DoseLevel-2%	DoseLevel-3%	DoseLevel-4%	DoseLevel-5%	DoseLevel-10%	DoseLevel-15%	DoseLevel-20%	DoseLevel-25%	DoseLevel-50%	DoseLevel-100%
Voxel Spacing	0.97 × 0.97 × 1	Same	Same	Same	Same	Same	Same	Same	Same	Same	Same
kVp	120: 131,100: 107, 140: 26, 80: 10	Same	Same	Same	Same	Same	Same	Same	Same	Same	Same
Manufacturer	Siemens	Same	Same	Same	Same	Same	Same	Same	Same	Same	Same
Age (Years)	63.71 ± 15.88	Same	Same	Same	Same	Same	Same	Same	Same	same	Same
Recon.Diamter (cm)	500	Same	Same	Same	Same	Same	same	Same	Same	Same	Same
Patient Height (m)	1.70 ± 0.10	Same	Same	Same	Same	Same	Same	Same	Same	Same	Same
Patient Weight (Kg)	72.92 ± 16.80	Same	Same	Same	Same	Same	Same	Same	Same	Same	Same
Rotation Time (seconds)	0.5	Same	Same	Same	Same	Same	Same	Same	same	same	same
Mean Tube Current (mA)	1.44 ± 1.22	3.406 ± 2.41	5.03 ± 3.79	6.87 ± 5.03	9.29 ± 6.06	19.08 ± 12.11	28.86 ± 18.15	38.65 ± 24.19	48.44 ± 30.25	97.38 ± 60.50	195.84 ± 120.82
Minimum Tube Current (mA)	0.41 ± 0.65	1.265 ± 1.26	2.05 ± 1.98	2.89 ± 2.61	3.91 ± 2.99	8.38 ± 6.019	12.79 ± 9.04	17.24 ± 12.09	21.71 ± 15.10	43.89 ± 30.19	88.46 ± 60.40
Maximum Tube Current (mA)	2.81 ± 1.96	6.04 ± 3.80	8.69 ± 5.98	11.74 ± 7.90	15.96 ± 9.69	32.40 ± 19.27	48.84 ± 28.82	65.30 ± 38.42	81.76 ± 48.13	164.01 ± 96.18	329.61 ± 191.81
SD of Tube Current (mA)	0.72 ± 0.49	1.36 ± 0.95	1.91 ± 1.53	2.52 ± 2.03	3.33 ± 2.45	6.63 ± 4.89	9.93 ± 7.34	13.23 ± 9.79	16.54 ± 12.26	33.08 ± 24.51	66.39 ± 48.94
CTDI_vol_ (mGy)	0.06 ± 0.05	0.14 ± 0.10	0.23 ± 0.17	0.31 ± 0.22	0.39 ± 0.26	0.80 ± 0.52	1.21 ± 0.78	1.62 ± 1.04	2.04 ± 1.30	4.10 ± 2.61	8.24 ± 5.21
Image Size	512 × 512	same	same	same	same	same	same	same	same	same	same
Number of Slices	661.33 ± 283.39	same	same	same	same	same	same	same	same	same	same

### Benchmarking the performance on simulated low-dose CT data

The FD-nnU-Net models were tested on all reconstructed CT images, including FD-CT and LD-CTs from 1 to 50% (11 cohorts of low-dose CT images) to delineate 21 defined organs. The model performance was evaluated in terms of well-established image segmentation metrics by comparing the predicted segmentation masks from LD-CT images to the reference masks generated from FD-CT images. Segmentation metrics including Dice coefficient, Jaccard, mean surface distance (MSD, mm), Hausdorff distance (HD, mm), and segment volume change (VD, mL) was calculated. This step was performed to assess the robustness of the nnU-Net trained model on a large dataset by simulating reduced CT image quality through lowering tube currents in low-dose and ultra-low-dose CT (ULD-CT) images. In addition, publicly available models, known as MOOSE^1^ [5], were inferenced on all dose levels using the same approach as our FD-nnU-Net models. MOOSE segmentations generated on FD-CT images were considered as the reference segmentation mask to benchmark its performance drop when shifting from a high-dose image to a low-dose image.

### Training the ULDCT segmentation models

According to the drop pattern in the segmentation performance of FD-nnU-Net models on ULD-CT images for different organs calculated in the former step, three image cohorts including LD-CT-1%, LD-CT-2%, and LD-CT-5% were used to train new nnU-Net segmentation models. The FD-CT generated masks using FD-nnU-Net model were considered as standard of reference segmentation. The training dataset was augmented using a realistic data augmentation approach by reconstructing images using two different algorithms implemented on Siemens CT scanner, namely ADMIRE and Filtered Back Projection (FBP), at slice thicknesses of 1 and 2 mm. This resulted in 12 samples per patient, from three dose levels (1%, 2%, and 5%) × two reconstruction algorithms (ADMIRE and FBP) × two slice thickness (1- and 2- mm).

In total, 21 organs categorized in two groups, namely bony structures (six organs including clavicles, femoral heads, hips, sacrum, rib cage, and vertebrae) and soft-tissue organs (15 organs including adrenal glands, aorta, brain, colon, eyeballs, GB, kidneys, liver, lungs, pancreas, erector spinae, small bowel, spleen, stomach, UB, and heart), were used to train two different models for two different tasks. We have developed an analytical image processing algorithm for segmenting body contours on CT images used in previous studies [9, 38, 39]. The algorithm consists of multiple 2D and 3D object detection tools using shape properties and Hounsfield unit (HU) values of the detected objects, hole filling, and removal of objects under the patient’s bed or smaller than a certain size. Reliable performance was achieved on FD-CT images. However, its response was inconsistent on LD-CT-X% images due to increased noise and changes in HU values. We defined the third task as segmenting the body contour on LD-CT images using the same group of datasets. The body contour mask is useful for calculation of water-equivalent diameter or other applications requiring patient size and attenuation parameters as well as cropping to foreground for accelerated DL inference.

**Fig.1:**
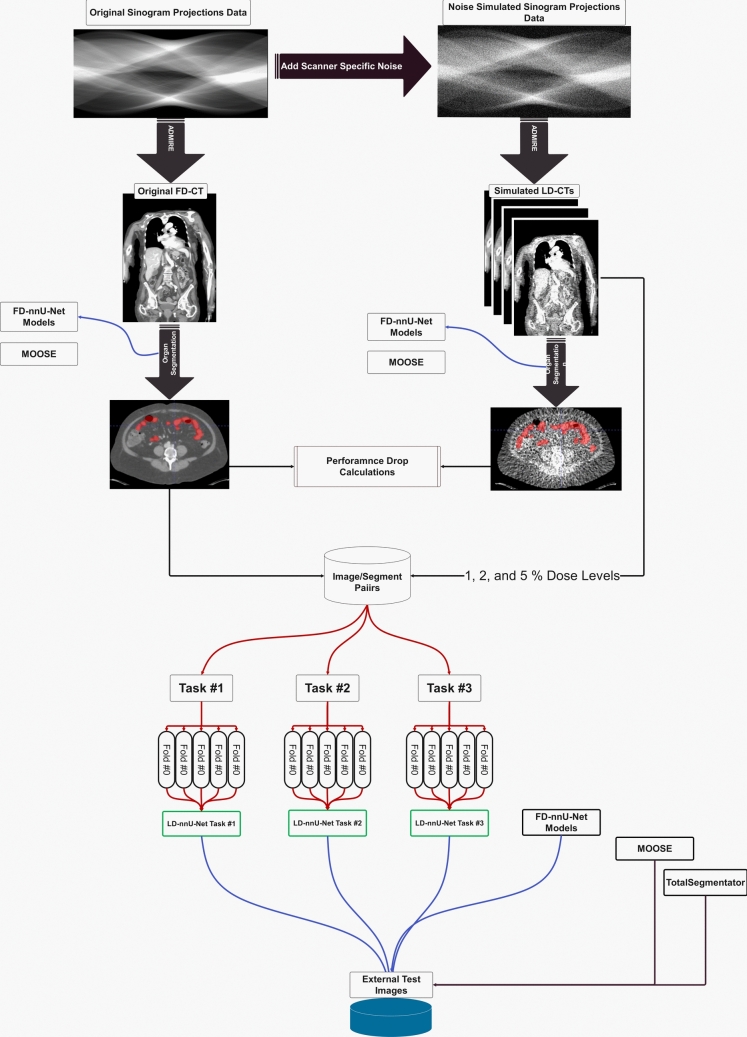
Steps followed in this study. The blue lines show inference of models whereas the red lines show training steps

**Fig. 2:**
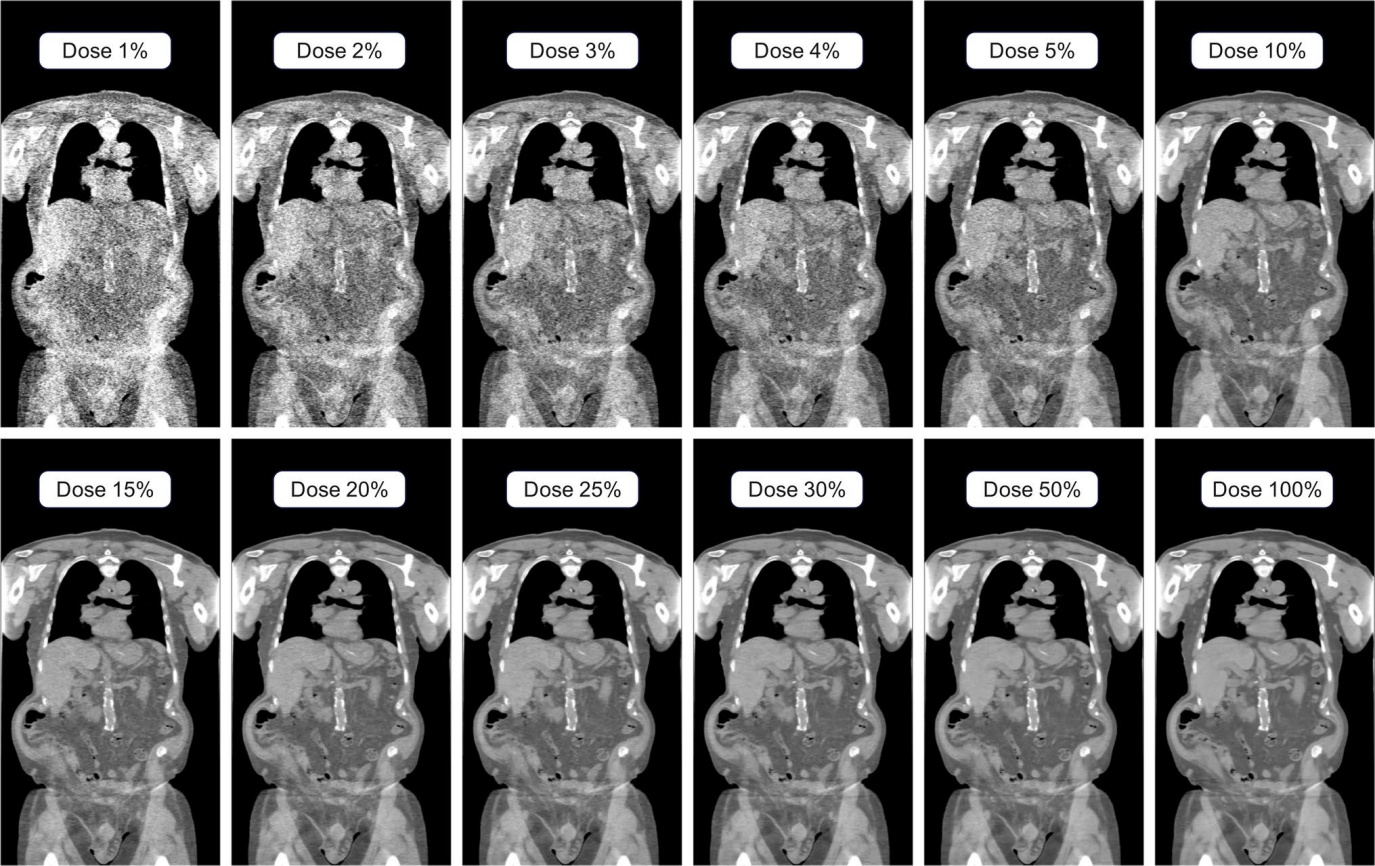
Example of low-dose CT images at different dose levels showing the pattern of noise in lower-dose simulated images. The same windowing of 600 Hus and level equal to zero was used for all images

nnU-Net training pipeline automatically selects the patch size, normalization values and network architecture according to image and segmentation characteristics. We separated bony structures from soft-tissues to allow the nnU-Net self-configuration algorithm to select the optimized hyperparameters specifically for each task. Three nnU-Net models were trained for bony organs (LD-nnU-Net task #1), soft-tissue organs (LD-nnU-Net task #2), and body contour (LD-nnU-Net task #3) using 274 × 12 image/segmentation pairs. nnU-Net version 2 using the default configuration for 3D high-resolution (3d_highres) training was used, including an initial learning rate of 1e-2, reduced each epoch with a decay of 3e-5, and cross-entropy loss function. The training length was extended from the default 1000 epochs to 2000 epochs to improve accuracy. Manual data split with fivefold cross-validation was implemented to ensure that all LD-CT-1%, LD-CT-2%, and LD-CT-5% with various reconstruction algorithms were in either the training or test set in each fold, preventing overfitting. In other words, with the fivefold data split, 80% of data were used for training and 20% for testing in each fold. The same image segmentation evaluation metrics used in the previous step were calculated for the models trained in fivefold cross-validation approach. Figure 1 presents the steps followed in this study whereas Fig. 2 depicts coronal images reconstructed using different radiation dose levels.

### External evaluation

To evaluate the models’ performance on external, unseen data, we validated our model on three local databases as well as two publicly available low-dose CT datasets, as described below. For the sake of reducing evaluation time, we selected a limited number of images from each database and conducted the visual inspection.

1. Dataset #1: Total-body CT images referred to our department for multiple myeloma indication. These images were acquired with 50 mA fixed tube current, a rotation time of 0.5 s (25 mAs), and 100 kVp, and reconstructed using a 50 cm field-of-view. The dataset included 34 CT images, 10 of these images were selected randomly.
2. Dataset #2: Chest and abdomen images of patients referred for ruling out Covid-19 during the pandemic. These images were acquired using tube current modulation, with a tube current of 20 to 50 mA and a rotation time of 0.5 s. This dataset consisted of 41 images, where 10 CT images were selected randomly.
3. Dataset #3: Chest CT images acquired for attenuation correction of cardiac SPECT images, referred to a private nuclear medicine center. The images were acquired using modulated tube currents from 10 to 40 mA. The dataset included 32 CT images where 10 of them were selected randomly.
4. Dataset #4: healthy whole-body PET/CT samples from Selfridge et al. study [33] acquired at 140 kVp and reference tube current of 5 mAs. This dataset corresponds to 20% dose level of our simulated dataset. It utilized a reference mAs of 5 mAs, which is calibrated for a patient with attenuation equivalent to a 20 cm round spherical phantom, i.e., higher tube current was applied for body parts with a water-equivalent diameter greater than 20 cm. The original published dataset included 30 images, of which 10 randomly selected cases were used in this study. It should be noted that while DL-generated segmentations provided by the authors were matched to standard-dose CT images acquired at 90 min, they are not co-registered with the low-dose CT images acquired at other time points.
5. Dataset #5: Chest and abdomen images from Grand Challenge 2016, shared by McCollough et al. [25]. The training dataset includes 10 pairs of standard-dose and simulated low-dose CT images. We included 5 images from the training dataset, which included both standard-dose and simulated low-dose images corresponding to one-quarter of the original radiation dose, with low-dose tube currents around 60 mA. These low-dose datasets were generated by adding Poisson noise to raw projection data and reconstructing the simulated noisy projections.

A total of 21 organs were delineated on these total-body images using both FD-nnU-Net and LD-nnU-Net models, and the segmentations were visualized using an open-source image visualization software. The images were visualized using commercial ITK-Snap software to visually compare segmentation masks generated by FD-nnU-Net and LD-nnU-Net models. Two publicly available models, namely MOOSE and models developed by Wasserthal et al. [17], known as TotalSegmentator,^2^ were used to segment the same organs on external datasets. The segmentation masks generated by these two public models compared with FD-nnU-Net and LD-nnU-Net models’ output. The segmentation masks of paired organs were unified, for example if they generate two separate segmentation masks for left and right clavicle, we considered both as a single organ for comparison with our models.

### Statistical evaluation

First, Kolmogorov Smirnov (KS) test was used to test the normality of data distributions. Then, according to results of KS test, Spearman correlation test was used seeking for correlation between the FD-nnU-Net performance on simulated low-dose and ultra-low-dose CT images with patients and acquisition parameters of patients’ including weight, size, BMI, and acquisition kVp. The Wilcoxon test was used to compare the performance of LD-nnU-Net models on images with different reconstructions, slice thickness, and dose levels in fivefold cross-validation data split. Two-tailed P-values less than 0.05 were considered as statistically significant.

## Results

### Benchmarking performance on low-dose CT data

**Fig. 3:**
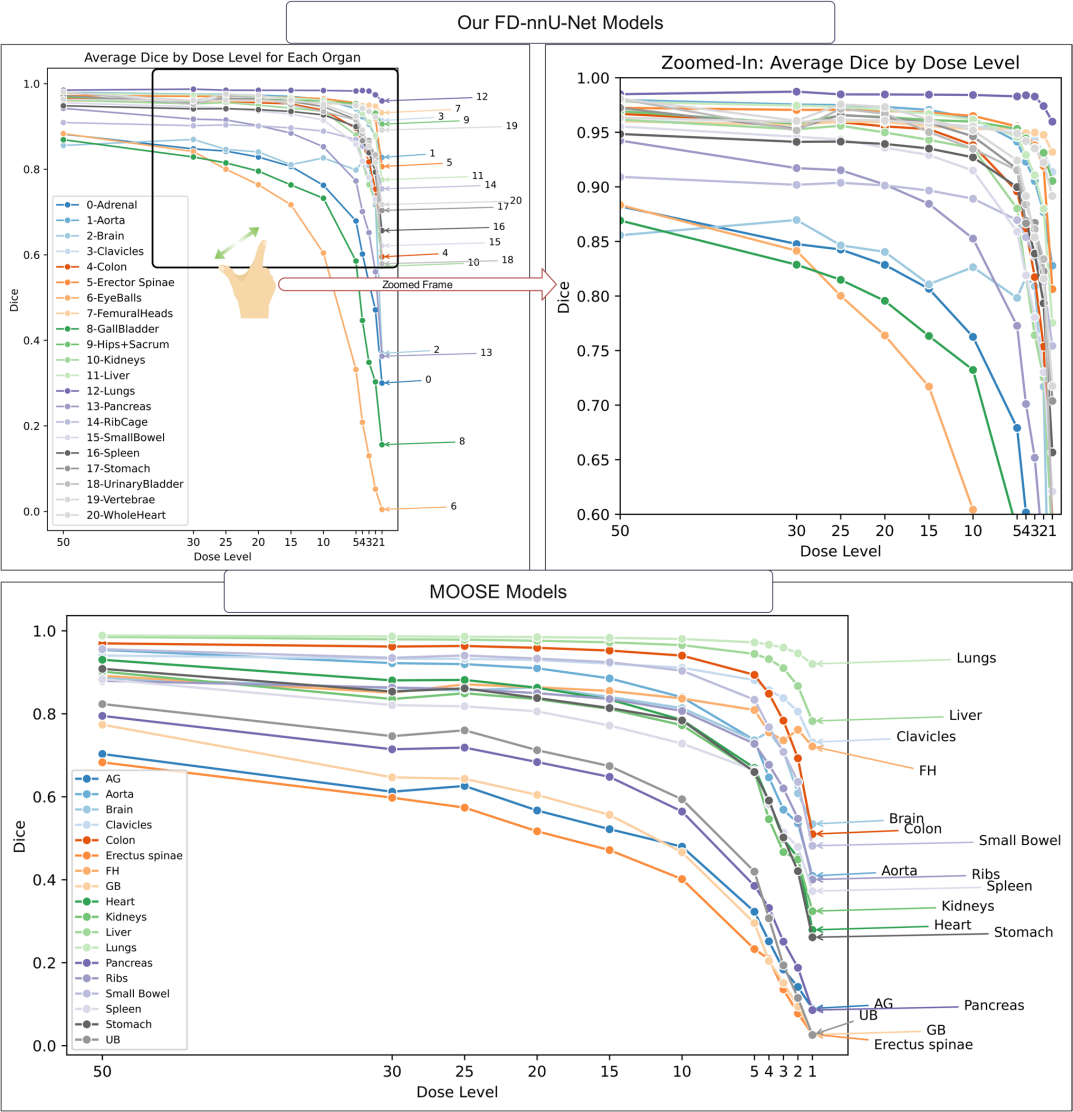
Plots showing the pattern of the Dice coefficient as a function of the dose level. The lines show the organ number summarized in the legend for simple visualization. The top row shows the Dice coefficient for our FD-nnU-Net whereas the button row shows the performance of MOOSE. The upper right image is the magnified version of changes in our FD-nnU-Net behavior for readability. The figure legend provides the meaning of line colors

This section describes the pattern of changes observed in the FD-nnU-Net segmentation models when tested on low-dose images. It aims to understand the model’s limitations and estimate the reduction in segmentation accuracy across different dose levels. Figure 3 shows the drop in the Dice coefficient observed on the plots for all organs generated by using low-dose images as input to FD-nnU-Net models. Table 2 summarizes the mean and standard deviations of Dice metric for the mentioned configuration. The most significant decline in performance in terms of Dice metric was seen in eyeballs, with Dice < 0.10 on ULDCT images. This was followed by gall bladder and adrenal gland organs. While some organs, including those with high objective contrast, such as lungs and hips, were the least affected by dose level reduction. Using a 50% dose reduction resulted in Dice factors lower than 0.90 for the eyeballs, adrenal glands, gall bladder and brain. In contrast, the rest of organ segmentations had Dice > 0.90. The behavior of nnU-Net organ segmentation when changing dose levels is summarized in supplementary Table 3 for other segmentation metric such as MSD, HD and volume differences. In contrast, a more severe drop in the performance for MOOSE segmentation models inferenced on the simulated low-dose images was noticed (Fig. 3). For some organs, such as the adrenal gland, the Dice coefficient dropped to less than 0.70 even on 50% dose level CT images. The less severe drops were observed for the lungs, while a Dice coefficient of less than 0.80 was observed on 50% dose level CT images, reaching Dice less than 0.50 for the majority of organs when the dose level was less than 15% of the original standard-dose CT.

**Table 2:** Performance of organs segmentation using FD-nnU-Net models tested on low-dose CT images in terms of Dice coefficient

	Radiation Dose Level										
	1%	2%	3%	4%	5%	10%	15%	20%	25%	30%	50%
Organ	Dice (mean ± SD)										
AG	0.300 ± 0.276	0.471 ± 0.269	0.535 ± 0.254	0.602 ± 0.242	0.679 ± 0.209	0.763 ± 0.18	0.807 ± 0.143	0.829 ± 0.126	0.843 ± 0.118	0.848 ± 0.104	0.882 ± 0.081
Aorta	0.828 ± 0.128	0.877 ± 0.113	0.905 ± 0.071	0.923 ± 0.071	0.941 ± 0.051	0.963 ± 0.026	0.970 ± 0.017	0.973 ± 0.013	0.975 ± 0.013	0.975 ± 0.012	0.980 ± 0.010
Brain	0.370 ± 0.305	0.717 ± 0.384	0.809 ± 0.357	0.817 ± 0.361	0.798 ± 0.369	0.826 ± 0.339	0.811 ± 0.353	0.840 ± 0.329	0.846 ± 0.317	0.870 ± 0.301	0.856 ± 0.308
Clavicles	0.914 ± 0.080	0.932 ± 0.087	0.944 ± 0.042	0.947 ± 0.032	0.952 ± 0.031	0.955 ± 0.026	0.957 ± 0.026	0.959 ± 0.026	0.96 ± 0.026	0.957 ± 0.025	0.961 ± 0.025
Colon	0.595 ± 0.252	0.754 ± 0.199	0.817 ± 0.152	0.861 ± 0.135	0.896 ± 0.112	0.938 ± 0.075	0.953 ± 0.051	0.955 ± 0.065	0.959 ± 0.051	0.959 ± 0.060	0.967 ± 0.027
Eyeballs	0.005 ± 0.030	0.052 ± 0.177	0.13 ± 0.248	0.208 ± 0.306	0.332 ± 0.352	0.604 ± 0.345	0.717 ± 0.302	0.764 ± 0.281	0.800 ± 0.260	0.841 ± 0.225	0.883 ± 0.162
FH	0.932 ± 0.081	0.948 ± 0.080	0.95 ± 0.047	0.949 ± 0.05	0.954 ± 0.065	0.953 ± 0.081	0.956 ± 0.074	0.959 ± 0.073	0.960 ± 0.073	0.957 ± 0.046	0.96 ± 0.074
GB	0.156 ± 0.247	0.303 ± 0.355	0.349 ± 0.364	0.446 ± 0.371	0.586 ± 0.371	0.732 ± 0.318	0.763 ± 0.312	0.796 ± 0.288	0.815 ± 0.276	0.829 ± 0.244	0.869 ± 0.208
Sacrum	0.887 ± 0.089	0.924 ± 0.116	0.924 ± 0.066	0.938 ± 0.117	0.932 ± 0.082	0.939 ± 0.135	0.940 ± 0.085	0.949 ± 0.112	0.953 ± 0.035	0.953 ± 0.110	0.959 ± 0.065
Hips	0.961 ± 0.096	0.964 ± 0.033	0.958 ± 0.116	0.966 ± 0.044	0.962 ± 0.093	0.967 ± 0.040	0.964 ± 0.081	0.962 ± 0.072	0.954 ± 0.132	0.968 ± 0.041	0.970 ± 0.051
Kidneys	0.573 ± 0.390	0.725 ± 0.322	0.764 ± 0.288	0.818 ± 0.238	0.880 ± 0.186	0.935 ± 0.089	0.943 ± 0.094	0.950 ± 0.076	0.956 ± 0.063	0.953 ± 0.071	0.962 ± 0.061
Liver	0.775 ± 0.246	0.880 ± 0.167	0.911 ± 0.136	0.929 ± 0.123	0.946 ± 0.110	0.962 ± 0.101	0.967 ± 0.096	0.970 ± 0.087	0.973 ± 0.076	0.974 ± 0.072	0.978 ± 0.073
Lungs	0.960 ± 0.090	0.974 ± 0.100	0.983 ± 0.067	0.984 ± 0.070	0.983 ± 0.084	0.984 ± 0.084	0.984 ± 0.084	0.985 ± 0.084	0.985 ± 0.084	0.987 ± 0.067	0.985 ± 0.085
Pancreas	0.363 ± 0.329	0.560 ± 0.313	0.652 ± 0.284	0.701 ± 0.266	0.773 ± 0.222	0.853 ± 0.163	0.884 ± 0.125	0.902 ± 0.112	0.915 ± 0.091	0.917 ± 0.091	0.942 ± 0.072
Erectus spinae	0.806 ± 0.152	0.919 ± 0.036	0.938 ± 0.021	0.948 ± 0.018	0.955 ± 0.017	0.965 ± 0.015	0.968 ± 0.015	0.970 ± 0.015	0.971 ± 0.016	0.971 ± 0.016	0.972 ± 0.016
Ribs	0.754 ± 0.114	0.813 ± 0.104	0.836 ± 0.089	0.854 ± 0.084	0.87 ± 0.075	0.889 ± 0.071	0.897 ± 0.071	0.901 ± 0.071	0.904 ± 0.071	0.902 ± 0.076	0.909 ± 0.072
Small Bowel	0.621 ± 0.269	0.730 ± 0.253	0.781 ± 0.234	0.819 ± 0.231	0.859 ± 0.204	0.915 ± 0.133	0.929 ± 0.127	0.936 ± 0.112	0.943 ± 0.106	0.946 ± 0.09	0.955 ± 0.085
Spleen	0.657 ± 0.328	0.793 ± 0.254	0.839 ± 0.219	0.869 ± 0.19	0.900 ± 0.155	0.927 ± 0.14	0.935 ± 0.131	0.939 ± 0.126	0.941 ± 0.124	0.941 ± 0.124	0.948 ± 0.117
Stomach	0.704 ± 0.277	0.823 ± 0.199	0.867 ± 0.157	0.867 ± 0.162	0.916 ± 0.107	0.946 ± 0.077	0.959 ± 0.071	0.963 ± 0.068	0.966 ± 0.067	0.953 ± 0.086	0.972 ± 0.064
UB	0.579 ± 0.314	0.834 ± 0.158	0.861 ± 0.127	0.884 ± 0.099	0.915 ± 0.094	0.935 ± 0.084	0.950 ± 0.066	0.967 ± 0.039	0.972 ± 0.034	0.952 ± 0.054	0.981 ± 0.024
Vertebrae	0.892 ± 0.062	0.922 ± 0.068	0.935 ± 0.027	0.942 ± 0.023	0.949 ± 0.021	0.955 ± 0.02	0.958 ± 0.02	0.961 ± 0.02	0.962 ± 0.021	0.959 ± 0.021	0.963 ± 0.021
Heart	0.718 ± 0.224	0.816 ± 0.204	0.854 ± 0.149	0.892 ± 0.116	0.924 ± 0.099	0.952 ± 0.049	0.962 ± 0.042	0.973 ± 0.035	0.976 ± 0.033	0.960 ± 0.035	0.981 ± 0.03

*AG* Adrenal gland, *GB* Gall Bladder, *FH* Femoral Heads, UB: Urinary Bladder

Supplementary Fig. 3 shows the number of segments with a Dice coefficient of less than 0.05, as well as the number of empty predicted segmentations where a specific organ is present in the reference segmentation mask, but FD-nnU-Net models predicted all voxels as zero in the predicted segmentation mask. The greatest number of empty segmentations and very low Dice values were observed for gall bladder and eyeballs. As the dose level increases, the number of images with Dice < 0.05 and empty predicted segmentation decreases for all organs. Supplementary Table 4 shows detailed information about these evaluations.

Supplementary Figs. 1 and 2 show the heatmaps of the Spearman test *P*-values for each organ included in task #1 and #2, respectively.

### ULDCT segmentation

An average Dice of 0.937 ± 0.049, 0.905 ± 0.117, and 0.984 ± 0.023 was achieved in the fivefold cross-validation data split for tasks #1, #2, and #3, respectively. Table 3 summarizes overall performance metrics of the LD-nnU-Net models for all three tasks using fivefold cross-validation data split. It should be noted that the segmentation performance was consistent over changes of reconstruction algorithm (FBP vs ADMIRE), dose level (1%, 2%, and 5%) and slice thickness (1 and 2 mm) according to the P-value of Wilcoxon test, which was >  > 0.05 for all comparisons.

**Table 3:** Overall performance of LD-nnU-Net models in fivefold cross-validation. mean ± SD (minimum–maximum)

Task	Segment–subtype	Dice coefficient	Jaccard distance	Mean Surface Distance	Volume Difference
Task #1	Clavicles	0.941 ± 0.042 (0.596–0.983)	0.891 ± 0.064 (0.424- 0.966)	1.226 ± 5.526 (0.037–58.115)	0.900 ± 5.078 (− 8.403–57.250)
	Femoral Heads	0.956 ± 0.046 (0.500–0.991)	0.919 ± 0.069 (0.334–0.982)	2.436 ± 8.312 (0.058–70.065)	4.602 ± 52.710 (− 177.872–771.979)
	Hips	0.947 ± 0.056 (0.045–0.980)	0.902 ± 0.063 (0.023–0.960)	0.460 ± 1.636 (0.106–31.924)	1.533 ± 5.009 (− 9.004–34.431)
	Ribs	0.943 ± 0.021 (0.840–0.976)	0.893 ± 0.036 (0.723–0.953)	0.343 ± 0.419 (0.095–3.302)	0.73 ± 14.257 (− 28.813–97.916)
	Sacrum	0.888 ± 0.04 (0.766–0.960)	0.801 ± 0.065 (0.620–0.923)	0.349 ± 0.362 (0.064–4.622)	5.011 ± 7.432 (− 14.421–58.785)
	Vertebrae	0.965 ± 0.045 (0.105–0.987)	0.935 ± 0.05 (0.055–0.975)	0.390 ± 2.231 (0.060–44.072)	− 4.378 ± 35.060 (− 675.512–25.810)
Task #2	AG	0.701 ± 0.129 (0.069–0.903)	0.554 ± 0.141 (0.036–0.823)	1.289 ± 1.781 (0.274–20.803)	0.250 ± 1.090 (− 3.772–4.124)
	Aorta	0.948 ± 0.030 (0.653–0.981)	0.902 ± 0.050 (0.485–0.962)	0.480 ± 0.411 (0.114–6.356)	− 0.010 ± 10.869 (− 132.371–27.194)
	Brain	0.948 ± 0.136 (0.048–0.994)	0.922 ± 0.163 (0.024–0.989)	2.873 ± 9.156 (0.109–53.546)	− 6.352 ± 91.536 (− 1055.833–497.010)
	Colon	0.911 ± 0.060 (0.472–0.979)	0.841 ± 0.088 (0.309–0.959)	1.243 ± 1.116 (0.102–11.813)	− 3.227 ± 38.880 (− 198.931–199.265)
	Eyeballs	0.864 ± 0.129 (0.150–0.954)	0.777 ± 0.145 (0.081–0.911)	1.111 ± 2.160 (0.232–14.726)	0.706 ± 2.949 (− 3.937–15.450)
	GB	0.794 ± 0.201 (0.034–0.975)	0.695 ± 0.222 (0.017–0.950)	2.133 ± 3.937 (0.194–57.010)	0.167 ± 5.971 (− 33.752–28.901)
	Kidneys	0.934 ± 0.064 (0.418–0.989)	0.882 ± 0.091 (0.264–0.977)	0.859 ± 2.53 (0.091–39.908)	− 0.047 ± 14.634 (− 174.193–60.743)
	Liver	0.966 ± 0.054 (0.123–0.991)	0.939 ± 0.067 (0.066–0.982)	1.355 ± 5.794 (0.166–122.374)	17.562 ± 72.206 (− 414.515–904.794)
	Lungs	0.986 ± 0.047 (0.09–0.996)	0.974 ± 0.054 (0.047–0.993)	0.682 ± 8.151 (0.057–190.525)	1.018 ± 17.058 (− 108.727–51.277)
	Pancreas	0.838 ± 0.107 (0.074–0.959)	0.733 ± 0.136 (0.039–0.921)	1.438 ± 1.707 (0.256–17.747)	0.476 ± 6.622 (− 30.031–36.549)
	Erec− r Spinae	0.939 ± 0.019 (0.720–0.970)	0.886 ± 0.031 (0.563–0.942)	0.686 ± 0.232 (0.299–3.062)	− 2.733 ± 18.957 (− 161.4–58.876)
	Small Bowel	0.874 ± 0.111 (0.082–0.979)	0.789 ± 0.138 (0.043–0.959)	1.464 ± 1.959 (0.11–26.555)	− 1.534 ± 42.004 (− 180.399–178.74)
	Spleen	0.937 ± 0.098 (0.062–0.990)	0.893 ± 0.123 (0.032–0.981)	1.188 ± 3.58 (0.103–50.982)	4.302 ± 59.446 (− 931.292–448.654)
	Stomach	0.935 ± 0.058 (0.435–0.983)	0.882 ± 0.085 (0.278–0.966)	1.002 ± 1.544 (0.192–15.57)	− 1.258 ± 40.859 (− 649.098–103.585)
	UB	0.917 ± 0.068 (0.450–0.986)	0.854 ± 0.101 (0.291–0.973)	1.281 ± 2.069 (0.192–21.373)	1.421 ± 18.858 (− 74.136–201.738)
	Heart	0.965 ± 0.028 (0.419–0.984)	0.933 ± 0.039 (0.265–0.968)	2.824 ± 7.874 (0.302–57.468)	− 1.683 ± 32.332 (− 503.181–108.438)
Task #3	Body	0.984 ± 0.023 (0.867–0.999)	0.97 ± 0.042 (0.766–0.999)	2.861 ± 4.63 (0.029–23.573)	− 805.536 ± 2717.645 (− 17,809.644–1894.78)

**Fig. 4:**
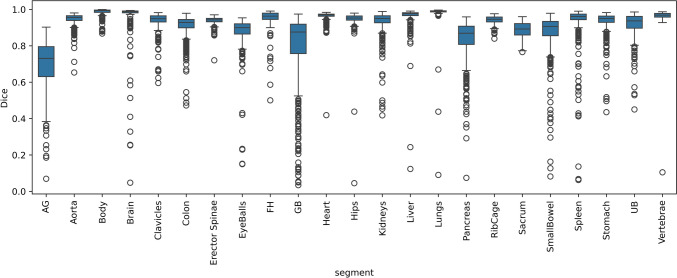
Box plot of Dice coefficients for the different segmented organs for fivefold cross-validation. The training was done on dose levels of 1%, 2%, and 5%

The highest Dice values were achieved for body, lungs, liver, vertebrae, and heart, while the lowest Dice values were observed for the adrenal glands. Figure 4 shows the box plot of Dice coefficients for the fivefold cross-validation.

**Fig. 5:**
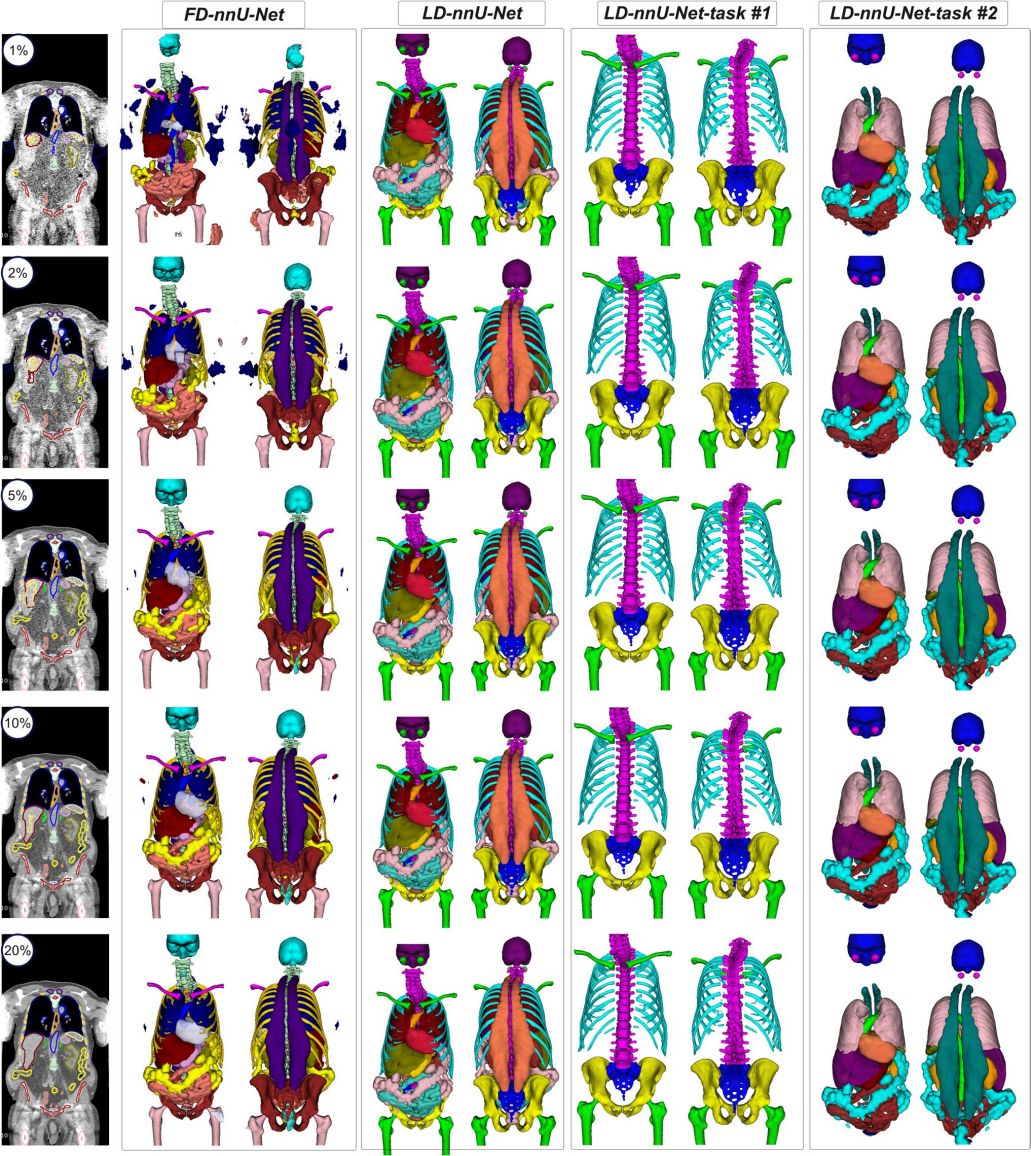
Representative segmentation examples of two FD and LD models tested on a 1% dose level CT image

Figure 5 presents examples of organs segmented by LD-nnU-Net models on 1% dose level CT images, compared to the output of FD-nnU-Net models. As presented in Fig. 5, the LD-nnU-Net models outperformed the available methods trained on standard-dose CTs, named FD-nnU-Net models.

### External evaluation

Supplementary Fig. 4 shows axial slices of images from external datasets showing the superiority of LD-nnU-Net model over FD versions. Supplementary Figs. 5 to 8 present examples of the aforementioned CT image for dataset #1 and #5, respectively. The high-resolution images are presented in editable format.

Dataset #5 was the only dataset providing co-registered standard-dose and low-dose CT images, with the low-dose corresponding to one-quarter (¼) the original standard-dose CT images. As a result, head-to-head comparisons were not possible for all images. Datasets #1 to #3 were only available in low-dose acquisitions, while dataset #4 included both high- and low-dose CT images, but they were not co-registered. After visual assessment, the performance of both FD and LD nnU-Net models was acceptable and the same applies to datasets #4 and #5. However, there were differences between these two models on datasets of #1, #2, and #3. Both LD and FD models did not perform well on dataset #3, which contained ultra-low-dose CT images with lower kVp and presented severe streak artifacts. However, the LD models outperformed the FD models.

The most frequent underperformance was observed in the segmentation of the gall bladder, aorta, adrenal glands, kidneys, and small bowel, which was consistent with the results of the previous step from testing FD-nnU-Net models on simulated low-dose CT images. The number of images with at least one organ segmented with errors by FD-nnU-Net but improved by LD-nnU-Net was 7/10,8/10, 10/10, 1/10, and 0/5 for datasets #1 to #5, respectively. This underperformance was more significant in patients with higher body habitus, arms down position, and when fixed tube current protocols were used. The fixed tube current mode means lower tube current for larger body sizes compared to modulated tube current acquisitions.

**Fig. 6:**
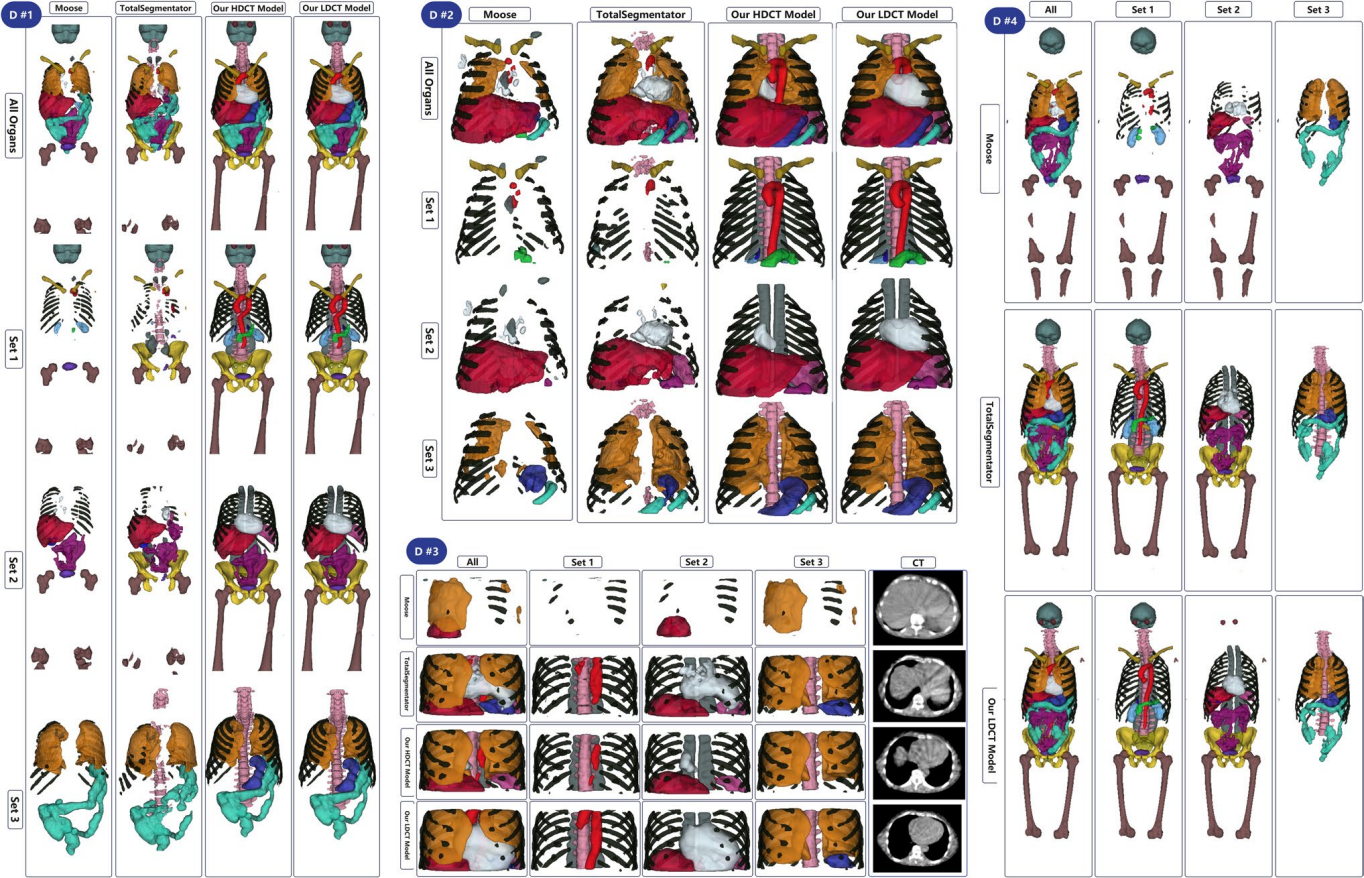
Segmentations generated by four models on four examples from datasets of #1 to #4 showing the excellent performance of our models on low-dose and ultra-low-dose CT images and the poor performance of other available models on low-dose CT images while showing good performance on high-dose CT images. Color guide: AG: Blue, Aorta: Bright Red, Brain: Teal, Clavicle: Golden Yellow, Colon: Aqua Green, Eyeballs: Burgundy, FH: Muted Red, GB: Bright Blue, Sacrum: Mustard Yellow, Hips: Bright Yellow, Kidneys: Sky Blue, Liver: Crimson Red, Lungs: Orange, Pancreas: Emerald Green, Erectus Spinae: Dark Teal, Ribs: Olive Green, Small Bowel: Magenta, Spleen: Fuchsia, Stomach: Royal Blue, UB: Violet, Vertebrae: Light Pink, Heart: Pale Blue. Dataset #4 example image id is body-cts-001 in the uExplorer dataset. It should be mentioned that MOOSE segmentations of the vertebrae, hips, and sacrum are not included due to technical issues during inference

Figure 6 compares the segmentation outputs of our FD-nnU-Net, LD-nnU-Net, MOOSE and TotalSegmentator on an example from external datasets of #1, #2, #4, and #4. Our FD-nnU-Net models significantly outperformed the two publicly available models, while the LD-nnU-Net models provide even better results than FD models. In depth comparison of our LD-nnU-Net models with MOOSE for all cases belonging to datasets #1, #2, #3, and #5 is presented in the supplementary material. The image id in the public dataset of #4 is presented on the supplementary images, which can be used for tracing the images.

Supplementary Fig. 9 shows an example of segmentation output for task #3 on a challenging case with the external object outside the patient’s body. While this could be problematic for analytic image processing algorithms that rely on object detection, the deep learning LD-nnU-Net model achieved promising results.

## Discussion

CT is the largest contributor to medical radiation dose to the public [40]. Various strategies were devised to reduce the CT radiation dose while maintaining acceptable image quality. However, CT images acquired with ultra-low-dose procedures suffer from multiple artifacts due to photon deprivation, beam hardening and low quantum mottle. Ultra-low-dose imaging is more common for SPECT and PET attenuation and scatter correction, especially in dynamic imaging and repeated scans. Accurate automated organ segmentation from LDCT images is critical for image quantification, radiomics studies, dosimetry evaluation, as well as kinetic modeling. However, available DL-based CT segmentation models bear inherent limitations when applied to populations different from the training data [34], such as ultra-low-dose images presenting with severe noise patterns.

CT dose reduction has been a focus during the last two decades [41]. Low-dose imaging is commonly used for SPECT and PET attenuation and scatter correction, particularly in dynamic imaging and repeated scans. Studies have demonstrated that even ultra-low-dose CT images are sufficient for attenuation correction in PET and SPECT imaging [26, 27, 42]. Recent deep learning models trained on high-dose CT mages are robust against changes in reconstruction algorithms, scanners, and the presence or absence of contrast media in the patients’ body. However, organ segmentation on low-dose CT images remains a challenge due to the reduced performance of standard deep learning approaches, which are typically trained on high-dose CT images, as shown in Fig. 3. Multiple time-point imaging is a standard practice in nuclear medicine and personalized dosimetry, such as in dynamic modeling and theranostics for 177Lu treatments, where multiple CT images are acquired after radiotracer injection. External datasets #3 and #4 serve as a good example of low-dose CT images acquired using a low-dose protocol for SPCET and PET imaging, respectively. Another key application of low-dose imaging is total-body imaging for multiple myeloma follow-up [29], which was the primary indication for external dataset #1. Additionally, low-dose imaging is particularly recommended during pregnancy to minimize radiation risks to the embryo or fetus, given their heightened sensitivity to radiation [43]. Dataset #2 illustrates this application. Organ segmentation plays a critical role in applications like image quantification, parametric modeling, personalized dosimetry, radiomic studies, and outcome prediction. Accurate segmentation ensures consistency in these workflows, enabling precise treatment planning and evaluation of therapeutic outcomes. In these scenarios, access to reliable organ segmentation tools is crucial. Organ segmentation tool dedicated for low-dose CT images could help bridge the gap in segmentation performance across varying dose levels. A robust segmentation framework applicable to these clinical contexts can significantly reduce intra- and inter-observer variability, streamline workflows, and save time, ultimately enhancing patient care and safety.

To the best of our knowledge, there is a lack of dedicated automated models for low-dose CT organ segmentation. We utilized previously trained nnU-Net models trained using a large dataset comprising both adult and pediatric images collected from extensive public sources and local datasets available to segment multiple organs. Unfortunately, we did not have access to paired, co-registered CT images of the same patients without patient motion, across different dose levels and reconstruction parameters. To do so, we used simulated datasets created using Siemens ReconCT software through adding scanner-specific noise to projection data and generated low-dose images through reconstruction. The same approach was adopted in previous studies [44–46]. Besides, we validated scanner-specific noise simulation method by scanning a cylindrical CATPHAN phantom as described in the supplementary material. In this study, we first evaluated the performance of state-of-the-art nnU-Net models, trained on the most extensive publicly available dataset for multiple organ segmentation (FD-nnU-Net), on simulated low-dose CT images across different dose levels. The drop in performance was evaluated using different metrics, including the Dice coefficient, Jaccard index, surface distances, and changes in organs volume.

According to our results, testing FD-nnU-Net models on different dose levels showed a performance drop after a certain magnitude of tube current reduction. This drop varies depending on the organ being delineated, which is related to factors, such as organ’s shape, contrast with surrounding tissues, texture, and organs’ size. These patterns of performance drop were consistent for the included segmentation metrics, except for organ volume difference, which varies depending on organ size. There was a significant relationship between the performance drop and patient characteristics, such as age, height (size), weight, BMI and the kVp of the scan, particularly for organs, such as the adrenal glands and heart. According to the drop in performance calculated in the first step, which was significant on ultra-low-dose CT images, we concluded developing a new dedicated nnU-Net model for organ segmentation on noisy CT images. Network architecture and nnU-Net selected hyperparameters as well as the trained models and inference instructions are provided in the lab’s GitHub page.

Three tasks were defined to leverage the strengths of the self-configurating nnU-Net training pipeline: bony organ segmentation, soft-tissue organ segmentation, and body contour segmentation. The results were outstanding on fivefold cross-validation, showing excellent performance on very low-dose images where the FD-nnU-Net models performed poorly. Besides, we tested our models on three local and two publicly available external datasets to evaluate and ensure the robustness and generalizability of our proposed dedicated LD-nnU-Net models. We tested both FD and LD models on external datasets to fairly compare the benefits of using ultra-low-dose dedicated CT segmentation models. Consistent with performance drop observed on simulated low-dose CT images, the LD-nnU-Net models outperformed the FD-nnU-NET models, especially on lower-dose CT images, such as those with fixed tube currents for corpulent patients. This superiority was more evident for organs included in task #2, which contains soft-tissue organs and organs with lower objective contrast, such as gall bladder, while it was less pronounced for bony structures and lungs. However, for very low-dose images, such as those in external dataset #3, errors in bony structures segmented by FD models were addressed by using LD models. The arms positioned beside the trunk can cause beam hardening and photo-deprivation causing artifacts on low-dose CT images, especially for lower kVps. These artifacts caused abdominal segmentation errors in dataset #1. Nevertheless, for datasets #4 and #5, which contained higher-dose CT images, both FD-nnU-Net and LD-nnU-Net models produced similarly acceptable output. We used terms of low-dose and ultra-low-dose for CTs with tube currents around 50 mAs and lower than 15 mAs, respectively. However, depending on the specific tasks those terms could refer to different dose levels.

The nnU-Net default augmentation employs conventional transformation techniques, such as image flipping, to create more robust models. However, these transformations cannot replicate the variability in images resulting from different reconstruction parameters, convolution kernels, and algorithms, although image patterns can change with changing the reconstruction [2, 3]. By leveraging access to raw projection data and Siemens reconstruction software, we incorporated four types of images: ADMIRE with 1 mm slice thickness, ADMIRE with 2 mm slice thickness, FBP with 1 mm slice thickness, and FBP with 2 mm slice thickness.

We have compared our developed models with two publicly available and widely used organ segmentation models developed using nnU-NET pipeline. Regarding the performance drop in organ delineation on the simulated low-dose and ultra-low-dose CT images, our previously developed FD-nnU-Net model showed a less severe reduction in terms of Dice coefficient on low-dose images compared to MOOSE models (Fig. 3). All four models were compared on our FD and LD nnU-Net models, MOOSE, and TotalSegmentator models based on nnU-NET with minimal amendment to the default configuration (e.g., number of epochs), so that the main difference in performance observed among these models is related to the training dataset. We included a large number of versatile training data for previously developed FD-nnU-Net models and used realistic low-dose and ultra-low-dose CT images for training our LD-nnU-Net and included meaningful data augmentation to have a more robust algorithm. Iterative reconstruction algorithms, such as Siemens ADMIRE can provide better image quality compared to conventional FBP methods, especially on low-dose CT images. However, they are not widely available or common in clinical practice. Hence, we included FBP augmentation in our training dataset to ensure the generalizability of our models. It should be noted that MOOSE and TotalSegmentator disabled default flipping and mirroring of nnU-Net pipeline as they aimed to segment left and right organs separately. We included five external test sets from different low-dose CT imaging scenarios, including hybrid imaging for SPECT and PET, CT imaging of pregnant patients, serial total-body CT imaging and simulated low-dose images from previous studies. Our models showed significantly improved results compared to the other two publicly available models. Although we did not have access to the ground truth manual segmentation on the external datasets, the visual assessment of images shown in Fig. 6 demonstrates the excellent performance of our models where the other two models detected only a few organs. Supplementary figures of 5 to 8 show that our models outperformed MOOSE in all included cases in the external dataset and the performance was consistent among the different cases. In addition, we shared our trained models and inference instruction publicly (https://github.com/YazdanSalimi/Organ-Segmentation), to allow further validation of the findings.

Our study inherently bears few limitations. First, although we had a good amount of training data, including 274 CT images, a larger dataset would be better suited for deep learning tasks. In addition, all of them were acquired on Siemens scanners as Siemens reconstruction software was used to reconstruct the data. Secondly, we did not have access to reference segmentation masks on the external test datasets, which precluded quantitative assessment. However, we visualized all segmentations and compared our models’ output with publicly available organ segmentation models. Finally, we used simulated low-dose CT images as we did not have access to paired high-dose and low-dose CT images. Yet, excellent performance of our models on external test sets showed that the simulated data through scanner-specific calibration was realistic for training the segmentation models.

## Conclusion

We evaluated the behavior of deep learning-based CT organ segmentation models on ultra-low-dose CT images and concluded that there is a drop in the performance of those models trained on conventional CT images depending on the dose level and organ type. We trained new nnU-Net models dedicated to low-dose and ultra-low-dose CT images organ segmentation and tested them during cross-validation and external validation on five external datasets from other centers, reporting similar performance. These dedicated LD-nnU-Net models published for the first time can be used for automated segmentation on low-dose CT images for various indications.

## Supplementary Information

Below is the link to the electronic supplementary material.

Supplementary file1 (PDF 2929 kb)

## Data Availability

The data used in this work are not available. The models and inference codes are publicly available and shared on GitHub.

## Footnotes

1 https://github.com/ENHANCE-PET/MOOSE.

2 https://github.com/wasserth/TotalSegmentator
