# Supplementary material for: Deep learning-based segmentation of ultra-low-dose CT images using an optimized nnU-Net model
Source: Radiol Med. 2025 Mar 18;130(5):723–39. doi: 10.1007/s11547-025-01989-x (PMC12106562; doi:10.1007/s11547-025-01989-x)
Supplement: Supplementary file 1 — Supplementary file1 (PDF 2929 kb) [file 11547_2025_1989_MOESM1_ESM.pdf]

Supplementary material

Validation of simulated low-dose CT imaging

In this study, a Catphan 500 phantom was scanned using two different acquisition parameters. The first scan was performed with a tube voltage of 120 kVp, tube current of 100 mAs, and a pitch of 0.8. In the second scan, all parameters were kept constant except for the tube current, which was reduced to 10 mAs. The phantom's position remained unchanged between the scans. The raw projection data were collected and reconstructed using ReconCT software and ADMIRE reconstruction algorithm. the 10 mAs raw data reconstructed with standard ADMIRE reconstruction parameter, same as the rest of reconstruction in this study, while the 100 mAs CT raw projection data was reconstructed using noise simulation option activated corresponding to 10 % of the original raw data which corresponds to 10 mAs simulated CT. The real CT acquired at 10 mAs was compared with simulated 10 mAs CT by voxel-wise and segment level approaches. Cylindrical volumes of interest were drawn on multiple material in the CATPHAN phantom as well as uniform water area in the phantom, average and standard deviation of Hounsfield units were compared between the real and simulated 10 mAs images. In addition, voxel wise metrics of mean error (ME), mean absolute error (MAE), peak signal to noise ratio (PSNR), and structural similarity index (SSIM) were calculated between those two mentioned images.

Supplementary Table 1 shows the segment evaluations on the simulated low-dose image versus real low-dose acquired image.

Supplementary Table 1. Average and standard deviation of HUs inside every volumes of interest in CATPHAN phantom.

	**Real 10 mAs CT**		**Simulated 10 mAs CT**	
**Material**	**mean**	**std**	**mean**	**std**
**Air**	-685.3	147.9	-690.4	149.2
**PMP**	682.6	129.7	692.5	140.4
**Teflon**	77.7	211.9	78.1	213.8
**Delrin**	-57.7	10.1	-57.9	11.8
**uniform water**	13.3	6.4	15.2	8.4
**Acrylic**	-15.8	9.6	-16.2	9.5

Supplementary Table 2 shows the voxel-wised level evaluation comparing the real and simulated CT low-dose images.

Supplementary Table 2. Voxelwise metrics comparing the real and simulated low dose 10 mAs CT images.

**ME (HU)**	**MAE (HU)**	**RMSE (HU)**	**MSE (HU^2)**	**SSIM**	**PSNR**
0.55	6.83	0.55	0.30	99.07	43.49

Performance drop measurements

Supplementary table 3 summarizes the mean surface distance (MSD) of segmentations generated by FD-nnU-Net models inferenced on simulated low-dose images at different dose levels as well as HD and VD calculations.

Supplementary Table 3. Performance drop of FD-nnU-Net models tested on low-dose images in terms of mean surface distance.

organ	Dose Level										
**Organ**	**1**	**2**	**3**	**4**	**5**	**10**	**15**	**20**	**25**	**30**	**50**
					**Dice**						
**AG**	0.3 ± 0.276	0.471 ± 0.269	0.535 ± 0.254	0.602 ± 0.242	0.679 ± 0.209	0.763 ± 0.18	0.807 ± 0.143	0.829 ± 0.126	0.843 ± 0.118	0.848 ± 0.104	0.882 ± 0.081
**Aorta**	0.828 ± 0.128	0.877 ± 0.113	0.905 ± 0.071	0.923 ± 0.071	0.941 ± 0.051	0.963 ± 0.026	0.97 ± 0.017	0.973 ± 0.013	0.975 ± 0.013	0.975 ± 0.012	0.98 ± 0.01
**Brain**	0.37 ± 0.305	0.717 ± 0.384	0.809 ± 0.357	0.817 ± 0.361	0.798 ± 0.369	0.826 ± 0.339	0.811 ± 0.353	0.84 ± 0.329	0.846 ± 0.317	0.87 ± 0.301	0.856 ± 0.308
**Clavicles**	0.914 ± 0.08	0.932 ± 0.087	0.944 ± 0.042	0.947 ± 0.032	0.952 ± 0.031	0.955 ± 0.026	0.957 ± 0.026	0.959 ± 0.026	0.96 ± 0.026	0.957 ± 0.025	0.961 ± 0.025
**Colon**	0.595 ± 0.252	0.754 ± 0.199	0.817 ± 0.152	0.861 ± 0.135	0.896 ± 0.112	0.938 ± 0.075	0.953 ± 0.051	0.955 ± 0.065	0.959 ± 0.051	0.959 ± 0.06	0.967 ± 0.027
**Eyeballs**	0.005 ± 0.03	0.052 ± 0.177	0.13 ± 0.248	0.208 ± 0.306	0.332 ± 0.352	0.604 ± 0.345	0.717 ± 0.302	0.764 ± 0.281	0.8 ± 0.26	0.841 ± 0.225	0.883 ± 0.162
**Femoral Head**	0.932 ± 0.081	0.948 ± 0.08	0.95 ± 0.047	0.949 ± 0.05	0.954 ± 0.065	0.953 ± 0.081	0.956 ± 0.074	0.959 ± 0.073	0.96 ± 0.073	0.957 ± 0.046	0.96 ± 0.074
**GB**	0.156 ± 0.247	0.303 ± 0.355	0.349 ± 0.364	0.446 ± 0.371	0.586 ± 0.371	0.732 ± 0.318	0.763 ± 0.312	0.796 ± 0.288	0.815 ± 0.276	0.829 ± 0.244	0.869 ± 0.208
**Sacrum**	0.887 ± 0.089	0.924 ± 0.066	0.932 ± 0.082	0.94 ± 0.085	0.953 ± 0.035	0.959 ± 0.065	0.964 ± 0.033	0.966 ± 0.044	0.967 ± 0.04	0.962 ± 0.072	0.968 ± 0.041
**Hips**	0.924 ± 0.116	0.938 ± 0.117	0.939 ± 0.135	0.949 ± 0.112	0.953 ± 0.11	0.961 ± 0.096	0.958 ± 0.116	0.962 ± 0.093	0.964 ± 0.081	0.954 ± 0.132	0.97 ± 0.051
**Kidneys**	0.573 ± 0.39	0.725 ± 0.322	0.764 ± 0.288	0.818 ± 0.238	0.88 ± 0.186	0.935 ± 0.089	0.943 ± 0.094	0.95 ± 0.076	0.956 ± 0.063	0.953 ± 0.071	0.962 ± 0.061
**Liver**	0.775 ± 0.246	0.88 ± 0.167	0.911 ± 0.136	0.929 ± 0.123	0.946 ± 0.11	0.962 ± 0.101	0.967 ± 0.096	0.97 ± 0.087	0.973 ± 0.076	0.974 ± 0.072	0.978 ± 0.073
**Lungs**	0.96 ± 0.09	0.974 ± 0.1	0.983 ± 0.067	0.984 ± 0.07	0.983 ± 0.084	0.984 ± 0.084	0.984 ± 0.084	0.985 ± 0.084	0.985 ± 0.084	0.987 ± 0.067	0.985 ± 0.085
**Pancreas**	0.363 ± 0.329	0.56 ± 0.313	0.652 ± 0.284	0.701 ± 0.266	0.773 ± 0.222	0.853 ± 0.163	0.884 ± 0.125	0.902 ± 0.112	0.915 ± 0.091	0.917 ± 0.091	0.942 ± 0.072
**Erectus Spinae**	0.806 ± 0.152	0.919 ± 0.036	0.938 ± 0.021	0.948 ± 0.018	0.955 ± 0.017	0.965 ± 0.015	0.968 ± 0.015	0.97 ± 0.015	0.971 ± 0.016	0.971 ± 0.016	0.972 ± 0.016
**Ribs**	0.754 ± 0.114	0.813 ± 0.104	0.836 ± 0.089	0.854 ± 0.084	0.87 ± 0.075	0.889 ± 0.071	0.897 ± 0.071	0.901 ± 0.071	0.904 ± 0.071	0.902 ± 0.076	0.909 ± 0.072
**Small Bowel**	0.621 ± 0.269	0.73 ± 0.253	0.781 ± 0.234	0.819 ± 0.231	0.859 ± 0.204	0.915 ± 0.133	0.929 ± 0.127	0.936 ± 0.112	0.943 ± 0.106	0.946 ± 0.09	0.955 ± 0.085
**Spleen**	0.657 ± 0.328	0.793 ± 0.254	0.839 ± 0.219	0.869 ± 0.19	0.9 ± 0.155	0.927 ± 0.14	0.935 ± 0.131	0.939 ± 0.126	0.941 ± 0.124	0.941 ± 0.124	0.948 ± 0.117
**Stomach**	0.704 ± 0.277	0.823 ± 0.199	0.867 ± 0.157	0.867 ± 0.162	0.916 ± 0.107	0.946 ± 0.077	0.959 ± 0.071	0.963 ± 0.068	0.966 ± 0.067	0.953 ± 0.086	0.972 ± 0.064
**UB**	0.579 ± 0.314	0.834 ± 0.158	0.861 ± 0.127	0.884 ± 0.099	0.915 ± 0.094	0.935 ± 0.084	0.95 ± 0.066	0.967 ± 0.039	0.972 ± 0.034	0.952 ± 0.054	0.981 ± 0.024
**Vertebrae**	0.892 ± 0.062	0.922 ± 0.068	0.935 ± 0.027	0.942 ± 0.023	0.949 ± 0.021	0.955 ± 0.02	0.958 ± 0.02	0.961 ± 0.02	0.962 ± 0.021	0.959 ± 0.021	0.963 ± 0.021
**Heart**	0.718 ± 0.224	0.816 ± 0.204	0.854 ± 0.149	0.892 ± 0.116	0.924 ± 0.099	0.952 ± 0.049	0.962 ± 0.042	0.973 ± 0.035	0.976 ± 0.033	0.96 ± 0.035	0.981 ± 0.03
					**Jaccard**						
**AG**	0.21 ± 0.21	0.347 ± 0.222	0.403 ± 0.223	0.468 ± 0.219	0.546 ± 0.203	0.642 ± 0.184	0.695 ± 0.161	0.722 ± 0.143	0.742 ± 0.137	0.746 ± 0.125	0.796 ± 0.101
**Aorta**	0.724 ± 0.164	0.795 ± 0.144	0.834 ± 0.105	0.863 ± 0.1	0.893 ± 0.078	0.929 ± 0.045	0.943 ± 0.031	0.948 ± 0.024	0.951 ± 0.024	0.952 ± 0.023	0.96 ± 0.018
**Brain**	0.273 ± 0.252	0.671 ± 0.377	0.787 ± 0.361	0.801 ± 0.361	0.778 ± 0.372	0.804 ± 0.347	0.787 ± 0.358	0.82 ± 0.336	0.823 ± 0.328	0.851 ± 0.308	0.833 ± 0.32
**Clavicles**	0.848 ± 0.101	0.881 ± 0.099	0.896 ± 0.064	0.902 ± 0.054	0.911 ± 0.052	0.916 ± 0.046	0.919 ± 0.047	0.923 ± 0.047	0.924 ± 0.046	0.918 ± 0.045	0.926 ± 0.045
**Colon**	0.467 ± 0.243	0.639 ± 0.217	0.714 ± 0.18	0.775 ± 0.158	0.825 ± 0.137	0.89 ± 0.092	0.913 ± 0.07	0.919 ± 0.074	0.925 ± 0.065	0.924 ± 0.072	0.937 ± 0.045

**Eyeballs**	0.003 ± 0.017	0.039 ± 0.138	0.094 ± 0.189	0.159 ± 0.25	0.261 ± 0.298	0.511 ± 0.321	0.628 ± 0.295	0.681 ± 0.283	0.725 ± 0.27	0.771 ± 0.232	0.817 ± 0.183
**Femoral Head**	0.88 ± 0.095	0.907 ± 0.091	0.907 ± 0.071	0.907 ± 0.074	0.917 ± 0.083	0.917 ± 0.093	0.921 ± 0.089	0.928 ± 0.086	0.928 ± 0.086	0.92 ± 0.07	0.929 ± 0.087
**GB**	0.109 ± 0.184	0.241 ± 0.3	0.281 ± 0.321	0.367 ± 0.337	0.504 ± 0.347	0.656 ± 0.315	0.695 ± 0.311	0.729 ± 0.292	0.752 ± 0.281	0.76 ± 0.255	0.81 ± 0.23
**Sacrum**	0.804 ± 0.102	0.863 ± 0.073	0.879 ± 0.082	0.893 ± 0.084	0.913 ± 0.049	0.925 ± 0.069	0.932 ± 0.049	0.936 ± 0.058	0.938 ± 0.055	0.931 ± 0.079	0.941 ± 0.056
**Hips**	0.87 ± 0.112	0.896 ± 0.113	0.902 ± 0.132	0.915 ± 0.112	0.922 ± 0.111	0.934 ± 0.097	0.933 ± 0.116	0.936 ± 0.103	0.939 ± 0.094	0.929 ± 0.134	0.945 ± 0.065
**Kidneys**	0.504 ± 0.38	0.65 ± 0.325	0.686 ± 0.294	0.743 ± 0.254	0.819 ± 0.204	0.888 ± 0.116	0.902 ± 0.112	0.911 ± 0.096	0.92 ± 0.078	0.916 ± 0.088	0.93 ± 0.076
**Liver**	0.686 ± 0.267	0.813 ± 0.193	0.855 ± 0.16	0.884 ± 0.143	0.91 ± 0.127	0.937 ± 0.11	0.946 ± 0.105	0.951 ± 0.098	0.955 ± 0.09	0.956 ± 0.082	0.963 ± 0.085
**Lungs**	0.931 ± 0.101	0.959 ± 0.102	0.97 ± 0.068	0.973 ± 0.07	0.973 ± 0.084	0.976 ± 0.084	0.976 ± 0.084	0.977 ± 0.084	0.977 ± 0.084	0.979 ± 0.067	0.977 ± 0.084
**Pancreas**	0.277 ± 0.279	0.45 ± 0.286	0.54 ± 0.27	0.592 ± 0.26	0.67 ± 0.23	0.769 ± 0.183	0.809 ± 0.151	0.835 ± 0.138	0.853 ± 0.118	0.856 ± 0.115	0.897 ± 0.089
**Erectus Spinae**	0.697 ± 0.172	0.852 ± 0.055	0.884 ± 0.037	0.901 ± 0.032	0.915 ± 0.03	0.933 ± 0.029	0.938 ± 0.029	0.941 ± 0.029	0.943 ± 0.029	0.943 ± 0.03	0.947 ± 0.031
**Ribs**	0.617 ± 0.13	0.695 ± 0.118	0.727 ± 0.105	0.752 ± 0.099	0.775 ± 0.092	0.806 ± 0.089	0.818 ± 0.089	0.826 ± 0.09	0.83 ± 0.091	0.828 ± 0.094	0.839 ± 0.094
**Small Bowel**	0.497 ± 0.247	0.623 ± 0.247	0.685 ± 0.237	0.738 ± 0.231	0.79 ± 0.21	0.861 ± 0.152	0.884 ± 0.139	0.893 ± 0.132	0.905 ± 0.119	0.906 ± 0.107	0.923 ± 0.1
**Spleen**	0.568 ± 0.329	0.713 ± 0.268	0.767 ± 0.242	0.804 ± 0.218	0.843 ± 0.185	0.885 ± 0.162	0.897 ± 0.152	0.903 ± 0.148	0.906 ± 0.147	0.906 ± 0.148	0.917 ± 0.139
**Stomach**	0.602 ± 0.278	0.735 ± 0.219	0.79 ± 0.185	0.792 ± 0.193	0.859 ± 0.135	0.905 ± 0.101	0.927 ± 0.09	0.934 ± 0.083	0.939 ± 0.081	0.919 ± 0.104	0.951 ± 0.074
**UB**	0.471 ± 0.29	0.74 ± 0.185	0.773 ± 0.156	0.803 ± 0.132	0.854 ± 0.119	0.886 ± 0.105	0.911 ± 0.089	0.939 ± 0.06	0.947 ± 0.055	0.912 ± 0.083	0.963 ± 0.041
**Vertebrae**	0.809 ± 0.087	0.861 ± 0.081	0.88 ± 0.046	0.891 ± 0.04	0.904 ± 0.037	0.915 ± 0.037	0.921 ± 0.037	0.925 ± 0.037	0.927 ± 0.038	0.923 ± 0.038	0.93 ± 0.039
**Heart**	0.6 ± 0.235	0.729 ± 0.237	0.768 ± 0.185	0.82 ± 0.15	0.871 ± 0.13	0.912 ± 0.077	0.929 ± 0.069	0.95 ± 0.057	0.954 ± 0.055	0.926 ± 0.06	0.964 ± 0.05
**Sensitivity**											
**AG**	0.256 ± 0.267	0.42 ± 0.28	0.481 ± 0.27	0.551 ± 0.26	0.637 ± 0.237	0.725 ± 0.206	0.776 ± 0.171	0.797 ± 0.154	0.814 ± 0.146	0.819 ± 0.13	0.865 ± 0.1
**Aorta**	0.771 ± 0.177	0.837 ± 0.152	0.872 ± 0.109	0.9 ± 0.103	0.927 ± 0.079	0.957 ± 0.043	0.968 ± 0.027	0.972 ± 0.02	0.974 ± 0.019	0.974 ± 0.018	0.979 ± 0.013
**Brain**	0.275 ± 0.255	0.684 ± 0.373	0.807 ± 0.347	0.821 ± 0.356	0.815 ± 0.356	0.847 ± 0.322	0.829 ± 0.341	0.859 ± 0.308	0.876 ± 0.287	0.903 ± 0.262	0.895 ± 0.266
**Clavicles**	0.896 ± 0.106	0.923 ± 0.101	0.936 ± 0.058	0.941 ± 0.043	0.949 ± 0.04	0.953 ± 0.027	0.955 ± 0.031	0.959 ± 0.029	0.961 ± 0.025	0.953 ± 0.027	0.961 ± 0.025
**Colon**	0.481 ± 0.253	0.658 ± 0.224	0.736 ± 0.187	0.799 ± 0.163	0.85 ± 0.139	0.914 ± 0.091	0.937 ± 0.066	0.943 ± 0.072	0.949 ± 0.062	0.948 ± 0.07	0.962 ± 0.039
**Eyeballs**	0.003 ± 0.017	0.041 ± 0.146	0.095 ± 0.189	0.159 ± 0.252	0.265 ± 0.304	0.521 ± 0.33	0.647 ± 0.304	0.704 ± 0.294	0.752 ± 0.28	0.804 ± 0.241	0.866 ± 0.181
**Femoral Head**	0.938 ± 0.082	0.949 ± 0.078	0.959 ± 0.033	0.961 ± 0.031	0.963 ± 0.03	0.961 ± 0.075	0.968 ± 0.023	0.967 ± 0.023	0.968 ± 0.022	0.967 ± 0.024	0.968 ± 0.022
**GB**	0.111 ± 0.187	0.254 ± 0.316	0.301 ± 0.343	0.397 ± 0.362	0.532 ± 0.365	0.701 ± 0.326	0.747 ± 0.318	0.776 ± 0.299	0.803 ± 0.28	0.808 ± 0.259	0.864 ± 0.223
**Sacrum**	0.882 ± 0.113	0.932 ± 0.075	0.942 ± 0.084	0.949 ± 0.087	0.961 ± 0.04	0.964 ± 0.066	0.967 ± 0.039	0.968 ± 0.051	0.969 ± 0.048	0.964 ± 0.07	0.969 ± 0.047
**Hips**	0.953 ± 0.123	0.965 ± 0.12	0.968 ± 0.108	0.972 ± 0.087	0.977 ± 0.07	0.98 ± 0.015	0.975 ± 0.069	0.976 ± 0.063	0.976 ± 0.047	0.977 ± 0.014	0.977 ± 0.015
**Kidneys**	0.542 ± 0.412	0.685 ± 0.344	0.716 ± 0.308	0.774 ± 0.266	0.85 ± 0.212	0.92 ± 0.119	0.933 ± 0.114	0.942 ± 0.097	0.95 ± 0.078	0.946 ± 0.088	0.958 ± 0.073
**Liver**	0.734 ± 0.282	0.851 ± 0.196	0.892 ± 0.163	0.921 ± 0.143	0.947 ± 0.115	0.969 ± 0.096	0.974 ± 0.091	0.977 ± 0.083	0.978 ± 0.074	0.978 ± 0.078	0.98 ± 0.071
**Lungs**	0.972 ± 0.086	0.974 ± 0.101	0.982 ± 0.068	0.983 ± 0.07	0.982 ± 0.084	0.984 ± 0.084	0.985 ± 0.084	0.985 ± 0.084	0.985 ± 0.085	0.987 ± 0.067	0.985 ± 0.085
**Pancreas**	0.333 ± 0.343	0.516 ± 0.333	0.608 ± 0.304	0.674 ± 0.287	0.754 ± 0.25	0.852 ± 0.187	0.883 ± 0.15	0.902 ± 0.13	0.917 ± 0.104	0.918 ± 0.105	0.945 ± 0.075
**Erectus Spinae**	0.756 ± 0.195	0.909 ± 0.054	0.94 ± 0.026	0.953 ± 0.018	0.96 ± 0.017	0.97 ± 0.015	0.972 ± 0.015	0.973 ± 0.015	0.973 ± 0.016	0.973 ± 0.016	0.973 ± 0.016
**Ribs**	0.648 ± 0.137	0.726 ± 0.121	0.761 ± 0.106	0.791 ± 0.098	0.817 ± 0.087	0.859 ± 0.078	0.874 ± 0.074	0.882 ± 0.073	0.888 ± 0.072	0.891 ± 0.077	0.904 ± 0.072
**Small Bowel**	0.58 ± 0.282	0.695 ± 0.267	0.752 ± 0.245	0.793 ± 0.243	0.84 ± 0.216	0.902 ± 0.155	0.921 ± 0.14	0.932 ± 0.121	0.94 ± 0.112	0.939 ± 0.105	0.957 ± 0.077
**Spleen**	0.622 ± 0.341	0.763 ± 0.269	0.811 ± 0.24	0.847 ± 0.207	0.886 ± 0.174	0.923 ± 0.146	0.935 ± 0.129	0.94 ± 0.123	0.942 ± 0.124	0.943 ± 0.117	0.949 ± 0.115
**Stomach**	0.685 ± 0.279	0.799 ± 0.221	0.863 ± 0.167	0.908 ± 0.134	0.926 ± 0.113	0.958 ± 0.063	0.966 ± 0.053	0.968 ± 0.068	0.974 ± 0.038	0.97 ± 0.074	0.978 ± 0.054
**UB**	0.515 ± 0.312	0.79 ± 0.194	0.843 ± 0.158	0.876 ± 0.127	0.913 ± 0.114	0.943 ± 0.094	0.959 ± 0.074	0.972 ± 0.055	0.975 ± 0.051	0.962 ± 0.059	0.981 ± 0.038
**Vertebrae**	0.86 ± 0.087	0.902 ± 0.08	0.921 ± 0.037	0.932 ± 0.027	0.941 ± 0.024	0.952 ± 0.021	0.956 ± 0.02	0.959 ± 0.019	0.961 ± 0.019	0.96 ± 0.02	0.964 ± 0.02
**Heart**	0.711 ± 0.272	0.758 ± 0.25	0.805 ± 0.193	0.86 ± 0.154	0.903 ± 0.129	0.945 ± 0.066	0.956 ± 0.056	0.964 ± 0.053	0.968 ± 0.05	0.96 ± 0.039	0.977 ± 0.043

					**Specificity**						
**AG**	1.0 ± 0.0	1.0 ± 0.0	1.0 ± 0.0	1.0 ± 0.0	1.0 ± 0.0	1.0 ± 0.0	1.0 ± 0.0	1.0 ± 0.0	1.0 ± 0.0	1.0 ± 0.0	1.0 ± 0.0
**Aorta**	1.0 ± 0.0	1.0 ± 0.0	1.0 ± 0.0	1.0 ± 0.0	1.0 ± 0.0	1.0 ± 0.0	1.0 ± 0.0	1.0 ± 0.0	1.0 ± 0.0	1.0 ± 0.0	1.0 ± 0.0
**Brain**	1.0 ± 0.0	1.0 ± 0.0	1.0 ± 0.0	1.0 ± 0.001	1.0 ± 0.001	1.0 ± 0.001	1.0 ± 0.001	1.0 ± 0.001	1.0 ± 0.001	1.0 ± 0.001	1.0 ± 0.001
**Clavicles**	1.0 ± 0.0	1.0 ± 0.0	1.0 ± 0.0	1.0 ± 0.0	1.0 ± 0.0	1.0 ± 0.0	1.0 ± 0.0	1.0 ± 0.0	1.0 ± 0.0	1.0 ± 0.0	1.0 ± 0.0
**Colon**	1.0 ± 0.0	1.0 ± 0.0	1.0 ± 0.0	1.0 ± 0.0	1.0 ± 0.0	1.0 ± 0.0	1.0 ± 0.0	1.0 ± 0.0	1.0 ± 0.0	1.0 ± 0.0	1.0 ± 0.0
**Eyeballs**	1.0 ± 0.0	1.0 ± 0.0	1.0 ± 0.0	1.0 ± 0.0	1.0 ± 0.0	1.0 ± 0.0	1.0 ± 0.0	1.0 ± 0.0	1.0 ± 0.0	1.0 ± 0.0	1.0 ± 0.0
**Femoral Head**	1.0 ± 0.0	1.0 ± 0.0	1.0 ± 0.0	1.0 ± 0.0	1.0 ± 0.0	1.0 ± 0.0	1.0 ± 0.0	1.0 ± 0.0	1.0 ± 0.0	1.0 ± 0.0	1.0 ± 0.0
**GB**	1.0 ± 0.0	1.0 ± 0.0	1.0 ± 0.0	1.0 ± 0.0	1.0 ± 0.0	1.0 ± 0.0	1.0 ± 0.0	1.0 ± 0.0	1.0 ± 0.0	1.0 ± 0.0	1.0 ± 0.0
**Sacrum**	1.0 ± 0.0	1.0 ± 0.0	1.0 ± 0.0	1.0 ± 0.0	1.0 ± 0.0	1.0 ± 0.0	1.0 ± 0.0	1.0 ± 0.0	1.0 ± 0.0	1.0 ± 0.0	1.0 ± 0.0
**Hips**	1.0 ± 0.0	1.0 ± 0.0	1.0 ± 0.0	1.0 ± 0.0	1.0 ± 0.0	1.0 ± 0.0	1.0 ± 0.0	1.0 ± 0.0	1.0 ± 0.0	1.0 ± 0.0	1.0 ± 0.0
**Kidneys**	1.0 ± 0.0	1.0 ± 0.0	1.0 ± 0.0	1.0 ± 0.0	1.0 ± 0.0	1.0 ± 0.0	1.0 ± 0.0	1.0 ± 0.0	1.0 ± 0.0	1.0 ± 0.0	1.0 ± 0.0
**Liver**	0.999 ± 0.001	0.999 ± 0.001	0.999 ± 0.001	0.999 ± 0.001	1.0 ± 0.001	1.0 ± 0.001	1.0 ± 0.001	1.0 ± 0.001	1.0 ± 0.001	1.0 ± 0.001	1.0 ± 0.0
**Lungs**	0.999 ± 0.002	0.999 ± 0.003	1.0 ± 0.0	1.0 ± 0.0	1.0 ± 0.0	1.0 ± 0.0	1.0 ± 0.0	1.0 ± 0.0	1.0 ± 0.0	1.0 ± 0.0	1.0 ± 0.0
**Pancreas**	1.0 ± 0.0	1.0 ± 0.0	1.0 ± 0.0	1.0 ± 0.0	1.0 ± 0.0	1.0 ± 0.0	1.0 ± 0.0	1.0 ± 0.0	1.0 ± 0.0	1.0 ± 0.0	1.0 ± 0.0
**Erectus Spinae**	1.0 ± 0.0	1.0 ± 0.0	1.0 ± 0.0	1.0 ± 0.0	1.0 ± 0.0	1.0 ± 0.0	1.0 ± 0.0	1.0 ± 0.0	1.0 ± 0.0	1.0 ± 0.0	1.0 ± 0.0
**Ribs**	1.0 ± 0.0	1.0 ± 0.0	1.0 ± 0.0	1.0 ± 0.0	1.0 ± 0.0	1.0 ± 0.0	1.0 ± 0.0	1.0 ± 0.0	1.0 ± 0.0	1.0 ± 0.0	1.0 ± 0.0
**Small Bowel**	0.999 ± 0.001	1.0 ± 0.0	1.0 ± 0.0	1.0 ± 0.0	1.0 ± 0.0	1.0 ± 0.0	1.0 ± 0.0	1.0 ± 0.0	1.0 ± 0.0	1.0 ± 0.0	1.0 ± 0.0
**Spleen**	1.0 ± 0.001	1.0 ± 0.001	1.0 ± 0.001	1.0 ± 0.001	1.0 ± 0.001	1.0 ± 0.0	1.0 ± 0.0	1.0 ± 0.0	1.0 ± 0.0	1.0 ± 0.0	1.0 ± 0.0
**Stomach**	1.0 ± 0.001	1.0 ± 0.0	1.0 ± 0.0	1.0 ± 0.001	1.0 ± 0.0	1.0 ± 0.0	1.0 ± 0.0	1.0 ± 0.0	1.0 ± 0.0	1.0 ± 0.001	1.0 ± 0.0
**UB**	1.0 ± 0.0	1.0 ± 0.0	1.0 ± 0.0	1.0 ± 0.0	1.0 ± 0.0	1.0 ± 0.0	1.0 ± 0.0	1.0 ± 0.0	1.0 ± 0.0	1.0 ± 0.0	1.0 ± 0.0
**Vertebrae**	1.0 ± 0.0	1.0 ± 0.0	1.0 ± 0.0	1.0 ± 0.0	1.0 ± 0.0	1.0 ± 0.0	1.0 ± 0.0	1.0 ± 0.0	1.0 ± 0.0	1.0 ± 0.0	1.0 ± 0.0
**Heart**	0.999 ± 0.001	1.0 ± 0.0	1.0 ± 0.0	1.0 ± 0.0	1.0 ± 0.0	1.0 ± 0.0	1.0 ± 0.0	1.0 ± 0.0	1.0 ± 0.0	1.0 ± 0.0	1.0 ± 0.0
					**Mean Surface Distance**						
**AG**	8.158 ± 16.782	4.72 ± 7.962	4.062 ± 6.996	2.884 ± 6.609	1.802 ± 3.449	1.278 ± 3.344	0.823 ± 2.044	0.609 ± 1.415	0.546 ± 1.304	0.477 ± 1.002	0.272 ± 0.598
**Aorta**	2.953 ± 3.951	1.712 ± 2.713	1.113 ± 1.258	0.858 ± 1.136	0.581 ± 0.663	0.312 ± 0.275	0.225 ± 0.161	0.197 ± 0.121	0.184 ± 0.133	0.178 ± 0.111	0.137 ± 0.086
**Brain**	27.45 ± 69.244	12.835 ± 61.57	6.76 ± 32.351	18.786 ± 85.539	9.015 ± 41.186	7.926 ± 35.687	8.215 ± 34.281	7.265 ± 35.282	7.269 ± 34.771	6.402 ± 34.852	5.643 ± 32.195
**Clavicles**	0.771 ± 2.67	0.451 ± 1.55	0.455 ± 2.084	0.315 ± 1.02	0.239 ± 0.563	0.208 ± 0.599	0.182 ± 0.278	0.161 ± 0.219	0.147 ± 0.127	0.168 ± 0.145	0.143 ± 0.123
**Colon**	8.641 ± 8.938	4.647 ± 6.178	3.036 ± 3.87	2.157 ± 3.279	1.502 ± 2.259	0.728 ± 1.017	0.564 ± 1.015	0.797 ± 6.033	0.474 ± 1.118	0.423 ± 0.788	0.325 ± 0.42
**Eyeballs**	14.059 ± 0.438	11.197 ± 8.61	7.561 ± 7.26	8.069 ± 7.723	6.224 ± 7.067	2.448 ± 3.911	2.414 ± 4.552	2.048 ± 4.193	1.704 ± 4.074	1.385 ± 3.49	0.724 ± 1.662
**Femoral Head**	5.719 ± 14.467	1.657 ± 6.584	3.734 ± 11.399	4.938 ± 14.31	2.973 ± 11.301	3.216 ± 11.395	2.748 ± 10.638	1.271 ± 5.344	1.258 ± 5.287	3.718 ± 11.817	1.177 ± 5.138
**GB**	23.076 ± 52.966	15.8 ± 41.686	10.433 ± 21.918	7.756 ± 17.574	3.81 ± 7.54	2.923 ± 12.107	1.507 ± 3.496	1.116 ± 2.131	1.205 ± 3.631	1.052 ± 2.238	0.75 ± 1.795
**Sacrum**	1.02 ± 0.801	0.612 ± 0.338	0.494 ± 0.242	1.146 ± 8.378	0.311 ± 0.162	0.438 ± 2.804	0.212 ± 0.148	0.19 ± 0.138	0.182 ± 0.137	0.467 ± 3.108	0.17 ± 0.138
**Hips**	0.628 ± 0.372	2.474 ± 28.593	0.513 ± 1.184	0.368 ± 0.469	0.299 ± 0.359	0.329 ± 0.733	0.297 ± 0.64	0.222 ± 0.295	0.213 ± 0.265	0.393 ± 0.995	0.183 ± 0.174
**Kidneys**	12.985 ± 26.472	5.756 ± 13.932	3.731 ± 7.673	2.357 ± 5.038	1.354 ± 2.948	0.785 ± 3.054	0.658 ± 3.036	0.609 ± 3.007	0.539 ± 2.982	0.588 ± 3.248	0.478 ± 2.963
**Liver**	10.012 ± 17.073	4.262 ± 7.327	2.951 ± 5.165	2.028 ± 2.628	1.679 ± 4.158	1.237 ± 4.378	1.211 ± 4.737	1.023 ± 4.142	0.911 ± 3.787	0.796 ± 2.18	0.754 ± 3.535
**Lungs**	6.231 ± 20.787	2.222 ± 16.298	2.431 ± 28.923	2.418 ± 29.755	1.712 ± 24.175	0.855 ± 11.122	0.844 ± 11.18	0.839 ± 11.19	0.839 ± 11.184	1.014 ± 12.561	0.85 ± 11.42
**Pancreas**	16.089 ± 28.973	6.607 ± 10.852	4.084 ± 5.762	4.038 ± 7.586	2.421 ± 3.826	1.609 ± 4.371	1.277 ± 4.738	0.881 ± 1.956	0.87 ± 3.679	0.668 ± 1.66	0.455 ± 1.122

**Erectus Spinae**	3.968 ± 8.252	1.054 ± 1.698	0.699 ± 0.247	0.575 ± 0.225	0.477 ± 0.203	0.352 ± 0.186	0.315 ± 0.186	0.296 ± 0.186	0.285 ± 0.187	0.284 ± 0.189	0.266 ± 0.191
**Ribs**	1.777 ± 1.57	1.258 ± 6.634	0.693 ± 0.648	0.593 ± 0.559	0.432 ± 0.374	0.334 ± 0.351	0.28 ± 0.304	0.219 ± 0.135	0.208 ± 0.131	0.296 ± 0.422	0.187 ± 0.129
**Small Bowel**	6.722 ± 13.86	4.3 ± 11.556	2.688 ± 5.001	2.692 ± 7.29	2.039 ± 6.284	0.892 ± 3.007	0.741 ± 2.944	0.669 ± 2.731	0.643 ± 3.041	0.485 ± 1.82	0.403 ± 1.614
**Spleen**	16.631 ± 30.931	12.676 ± 39.029	7.15 ± 16.79	5.687 ± 15.794	4.189 ± 10.96	2.599 ± 9.377	2.524 ± 12.114	1.854 ± 6.38	1.414 ± 4.126	1.71 ± 6.934	1.31 ± 4.901
**Stomach**	29.126 ± 59.921	9.429 ± 30.047	6.945 ± 22.649	13.111 ± 31.5	3.661 ± 13.728	1.206 ± 3.156	0.494 ± 0.968	0.434 ± 0.855	0.368 ± 0.689	1.813 ± 5.438	0.296 ± 0.832
**UB**	9.633 ± 14.974	3.604 ± 10.393	5.073 ± 15.542	5.442 ± 15.903	3.062 ± 11.655	1.838 ± 6.296	1.702 ± 6.792	1.653 ± 9.855	0.738 ± 4.267	2.063 ± 7.246	0.611 ± 4.247
**Vertebrae**	0.841 ± 0.953	0.514 ± 0.597	0.434 ± 0.522	0.389 ± 0.503	0.317 ± 0.442	0.266 ± 0.429	0.245 ± 0.429	0.23 ± 0.429	0.226 ± 0.43	0.255 ± 0.477	0.216 ± 0.431
**Heart**	26.601 ± 25.352	9.088 ± 15.23	8.866 ± 12.247	6.844 ± 9.967	5.301 ± 11.253	3.933 ± 9.558	3.451 ± 9.314	2.46 ± 8.857	2.443 ± 9.148	3.366 ± 6.124	1.75 ± 8.12
**Hausdorff Distance**											
**AG**	inf ± nan	inf ± nan	inf ± nan	inf ± nan	inf ± nan	inf ± nan	4.55 ± 11.905	inf ± nan	2.994 ± 8.457	inf ± nan	inf ± nan
**Aorta**	22.729 ± 33.043	inf ± nan	7.711 ± 12.849	5.553 ± 9.942	3.59 ± 7.034	1.673 ± 2.508	1.224 ± 0.87	1.095 ± 0.547	1.066 ± 0.567	1.037 ± 0.43	0.926 ± 0.354
**Brain**	inf ± nan	inf ± nan	inf ± nan	inf ± nan	inf ± nan	inf ± nan	inf ± nan	inf ± nan	inf ± nan	inf ± nan	inf ± nan
**Clavicles**	6.57 ± 34.751	inf ± nan	4.101 ± 26.933	2.496 ± 17.046	1.649 ± 7.566	1.387 ± 7.525	1.032 ± 2.777	0.953 ± 2.52	0.792 ± 0.745	0.912 ± 0.806	0.783 ± 0.772
**Colon**	inf ± nan	inf ± nan	inf ± nan	inf ± nan	9.809 ± 19.092	inf ± nan	3.322 ± 9.823	2.937 ± 12.162	2.745 ± 9.515	2.182 ± 5.83	1.69 ± 5.11
**Eyeballs**	inf ± nan	inf ± nan	inf ± nan	inf ± nan	inf ± nan	inf ± nan	inf ± nan	inf ± nan	inf ± nan	inf ± nan	inf ± nan
**Femoral Head**	inf ± nan	inf ± nan	50.368 ± 160.491	55.258 ± 168.283	30.927 ± 129.22	inf ± nan	31.126 ± 128.851	15.664 ± 90.948	15.621 ± 90.817	47.625 ± 158.207	15.618 ± 90.824
**GB**	inf ± nan	inf ± nan	inf ± nan	inf ± nan	inf ± nan	inf ± nan	inf ± nan	inf ± nan	inf ± nan	inf ± nan	inf ± nan
**Sacrum**	inf ± nan	2.843 ± 2.871	inf ± nan	2.517 ± 8.724	1.36 ± 1.433	1.662 ± 6.963	1.134 ± 1.418	0.963 ± 0.355	0.925 ± 0.393	1.826 ± 8.226	0.809 ± 0.477
**Hips**	inf ± nan	inf ± nan	inf ± nan	inf ± nan	inf ± nan	1.238 ± 2.478	1.237 ± 2.389	1.069 ± 1.391	0.995 ± 1.258	1.451 ± 3.804	0.87 ± 1.13
**Kidneys**	inf ± nan	inf ± nan	inf ± nan	inf ± nan	inf ± nan	3.788 ± 14.789	2.674 ± 11.103	2.766 ± 11.911	2.304 ± 11.624	2.281 ± 11.954	1.818 ± 10.871
**Liver**	inf ± nan	inf ± nan	inf ± nan	inf ± nan	inf ± nan	inf ± nan	6.469 ± 18.421	5.417 ± 15.907	4.857 ± 14.914	5.311 ± 14.744	3.995 ± 14.848
**Lungs**	48.712 ± 77.381	inf ± nan	5.142 ± 37.674	3.82 ± 37.572	inf ± nan	inf ± nan	inf ± nan	inf ± nan	inf ± nan	2.532 ± 26.62	inf ± nan
**Pancreas**	inf ± nan	inf ± nan	inf ± nan	inf ± nan	inf ± nan	7.814 ± 17.456	inf ± nan	5.154 ± 15.329	4.558 ± 15.032	inf ± nan	inf ± nan
**Erectus Spinae**	20.179 ± 37.885	4.392 ± 11.571	2.446 ± 1.05	1.972 ± 0.941	1.583 ± 0.665	1.214 ± 0.374	1.146 ± 0.306	1.105 ± 0.296	1.084 ± 0.308	1.063 ± 0.336	0.994 ± 0.409
**Ribs**	inf ± nan	inf ± nan	inf ± nan	inf ± nan	inf ± nan	inf ± nan	inf ± nan	inf ± nan	inf ± nan	inf ± nan	inf ± nan
**Small Bowel**	inf ± nan	inf ± nan	inf ± nan	inf ± nan	inf ± nan	inf ± nan	inf ± nan	inf ± nan	2.947 ± 13.582	2.651 ± 13.532	2.158 ± 12.101
**Spleen**	inf ± nan	inf ± nan	inf ± nan	inf ± nan	inf ± nan	15.779 ± 47.13	14.303 ± 46.145	13.616 ± 42.855	10.187 ± 30.759	10.131 ± 30.999	8.385 ± 28.54
**Stomach**	inf ± nan	inf ± nan	32.709 ± 88.014	77.308 ± 144.431	31.334 ± 91.68	9.486 ± 41.149	2.864 ± 7.23	2.45 ± 5.6	2.122 ± 4.296	17.845 ± 68.564	1.94 ± 5.668
**UB**	inf ± nan	inf ± nan	34.29 ± 122.251	43.826 ± 144.664	23.192 ± 102.798	15.005 ± 71.369	17.338 ± 82.042	14.78 ± 86.853	8.08 ± 57.075	18.39 ± 83.675	7.619 ± 57.116
**Vertebrae**	5.674 ± 9.749	inf ± nan	3.305 ± 8.891	3.03 ± 8.871	2.366 ± 7.594	2.266 ± 7.611	2.226 ± 7.606	2.147 ± 7.616	2.098 ± 7.632	2.45 ± 8.504	2.018 ± 7.638
**Heart**	inf ± nan	inf ± nan	inf ± nan	60.072 ± 109.323	47.187 ± 107.077	34.673 ± 85.373	33.504 ± 91.028	22.837 ± 80.593	25.198 ± 88.945	36.138 ± 89.288	16.369 ± 67.962
**Volume Difference**											
**AG**	-3.352 ± 2.866	-1.994 ± 2.257	-1.593 ± 1.906	-1.191 ± 1.628	-0.812 ± 1.312	-0.529 ± 0.987	-0.383 ± 0.762	-0.367 ± 0.765	-0.312 ± 0.626	-0.327 ± 0.668	-0.157 ± 0.374
**Aorta**	-37.673 ± 45.923	-24.147 ± 33.564	-17.105 ± 22.195	-11.806 ± 18.939	-6.755 ± 14.07	-2.157 ± 7.531	-0.823 ± 4.927	-0.413 ± 3.677	-0.231 ± 3.276	-0.365 ± 3.145	-0.098 ± 2.155

**Brain**	-561.461 ± 471.248	-91.752 ± 231.605	-6.866 ± 103.697	7.281 ± 129.416	-3.33 ± 165.441	14.326 ± 117.43	18.592 ± 126.448	17.299 ± 133.541	17.293 ± 135.446	11.723 ± 123.171	14.85 ± 129.113
**Clavicles**	-1.822 ± 4.593	-0.962 ± 4.051	-0.542 ± 2.127	-0.537 ± 1.78	-0.245 ± 1.303	-0.244 ± 1.293	-0.212 ± 0.951	0.031 ± 0.825	0.033 ± 0.661	-0.338 ± 0.939	0.013 ± 0.64
**Colon**	-266.636 ± 212.456	-159.104 ± 146.355	-109.606 ± 118.024	-75.617 ± 81.165	-56.17 ± 60.689	-25.438 ± 30.66	-16.837 ± 22.36	-12.286 ± 17.913	-9.678 ± 15.494	-9.398 ± 16.053	-3.562 ± 12.105
**Eyeballs**	-14.872 ± 5.828	-14.628 ± 6.035	-14.406 ± 5.73	-13.483 ± 6.169	-11.039 ± 6.387	-6.595 ± 5.509	-4.507 ± 4.592	-3.355 ± 3.881	-2.421 ± 3.514	-2.032 ± 3.149	-0.769 ± 1.913
**Femoral Head**	6.412 ± 40.012	2.767 ± 44.15	10.975 ± 54.493	14.458 ± 56.457	7.825 ± 45.012	10.168 ± 45.87	9.38 ± 45.107	5.379 ± 43.377	5.345 ± 43.394	12.577 ± 52.772	5.242 ± 43.406
**GB**	-22.058 ± 19.403	-18.063 ± 19.087	-15.244 ± 17.774	-12.042 ± 15.389	-8.836 ± 13.17	-4.384 ± 8.827	-3.115 ± 7.669	-2.446 ± 6.952	-2.073 ± 6.879	-1.633 ± 4.435	-0.941 ± 5.173
**Sacrum**	-4.058 ± 26.472	3.323 ± 9.339	4.124 ± 7.261	3.787 ± 4.888	3.354 ± 3.491	2.254 ± 2.274	1.613 ± 2.048	1.182 ± 1.427	0.991 ± 1.288	1.064 ± 1.748	0.497 ± 0.965
**Hips**	45.777 ± 33.195	41.992 ± 19.521	34.069 ± 17.448	27.989 ± 14.351	23.593 ± 10.615	13.503 ± 7.072	10.669 ± 5.415	9.594 ± 4.453	7.975 ± 3.893	4.843 ± 4.207	3.773 ± 2.631
**Kidneys**	-71.909 ± 117.389	-43.582 ± 79.18	-39.738 ± 66.188	-28.751 ± 52.586	-16.096 ± 37.522	-6.05 ± 20.158	-3.661 ± 14.672	-2.656 ± 13.128	-1.908 ± 10.913	-1.93 ± 11.433	-1.033 ± 9.291
**Liver**	-310.732 ± 540.413	-159.857 ± 334.51	-104.013 ± 279.796	-56.869 ± 246.697	-17.706 ± 204.621	8.104 ± 170.817	8.188 ± 159.333	8.206 ± 141.944	6.785 ± 122.441	1.891 ± 129.679	-2.425 ± 102.068
**Lungs**	107.127 ± 316.424	0.436 ± 112.08	-8.44 ± 69.672	-7.051 ± 42.534	-4.777 ± 26.481	-0.155 ± 9.956	0.5 ± 6.884	0.657 ± 5.708	0.704 ± 5.019	1.111 ± 4.805	0.255 ± 4.153
**Pancreas**	-29.924 ± 31.829	-19.201 ± 25.077	-13.961 ± 20.988	-9.794 ± 20.363	-5.618 ± 16.008	-1.969 ± 11.784	-1.162 ± 9.316	-0.576 ± 6.813	-0.187 ± 5.617	-0.414 ± 5.265	0.245 ± 3.409
**Erectus Spinae**	-140.852 ± 179.82	-23.899 ± 48.397	-2.104 ± 27.143	4.951 ± 18.727	5.837 ± 14.208	7.604 ± 7.317	6.096 ± 5.656	4.927 ± 4.742	3.933 ± 4.034	3.264 ± 3.74	1.523 ± 3.151
**Ribs**	-90.405 ± 47.16	-67.264 ± 35.233	-55.327 ± 27.606	-45.112 ± 23.13	-36.259 ± 19.859	-20.088 ± 12.4	-14.601 ± 9.415	-12.457 ± 7.962	-9.918 ± 6.739	-6.629 ± 5.952	-3.448 ± 3.373
**Small Bowel**	-98.644 ± 191.497	-59.778 ± 148.203	-34.276 ± 92.165	-24.308 ± 65.418	-17.268 ± 76.937	-3.409 ± 36.852	-0.082 ± 27.82	0.434 ± 21.821	1.291 ± 19.32	2.724 ± 18.514	2.192 ± 15.463
**Spleen**	-63.599 ± 153.184	-38.091 ± 125.855	-30.065 ± 109.613	-20.07 ± 86.081	-11.155 ± 65.905	-6.691 ± 38.299	-4.28 ± 30.12	-3.781 ± 25.291	-3.113 ± 25.874	-3.589 ± 26.476	-3.229 ± 19.905
**Stomach**	-17.827 ± 156.921	-26.209 ± 103.892	-6.488 ± 90.202	24.791 ± 91.381	0.053 ± 50.871	3.705 ± 38.354	0.962 ± 23.39	0.996 ± 15.583	1.503 ± 15.207	7.696 ± 34.78	1.841 ± 11.739
**UB**	-56.907 ± 93.865	-20.855 ± 55.065	-12.542 ± 36.884	-9.861 ± 34.283	-4.055 ± 23.168	0.885 ± 21.644	0.938 ± 14.194	0.369 ± 11.807	0.305 ± 11.158	1.429 ± 9.632	-0.399 ± 6.442
**Vertebrae**	-47.738 ± 55.861	-30.141 ± 41.021	-19.031 ± 23.518	-13.293 ± 18.415	-10.916 ± 15.045	-4.049 ± 12.749	-2.275 ± 12.1	-1.945 ± 11.762	-1.017 ± 11.686	1.452 ± 13.196	1.092 ± 11.697
**Heart**	-91.041 ± 314.706	-159.206 ± 231.348	-116.557 ± 184.51	-71.422 ± 154.409	-48.261 ± 123.54	-12.721 ± 58.635	-9.931 ± 54.92	-16.447 ± 45.318	-14.058 ± 44.691	0.461 ± 45.906	-8.014 ± 36.794

Supplementary Table 4 presents the number of cases labeled as outlier in each dose level segmented by FD-nnU-Net models for three criteria of zero predicted volume (empty segmentation output), dice equal to zero, and dive less than 0.05.

Supplementary Table 4. Number of images in each dose level according to three different criteria.

Criteria	Organ	Dose-1%	Dose-2%	Dose-3%	Dose-4%	Dose-5%	Dose-10%	Dose-15%	Dose-20%	Dose-25%	Dose-30%	Dose-50%
Zero Predicted Volume	AG	75	58	19	10	6	6	1	4	2	1	1
	Brain	28	38	17	11	15	14	17	24	22	7	10
	Colon	8	6	1	2	0	1	0	0	0	0	0
	Eyeballs	83	77	52	40	38	13	6	5	3	3	1
	GB	124	100	67	43	48	23	25	20	15	12	8
	Heart	1	1	1	0	0	0	0	0	0	0	0
	Kidneys	35	20	9	4	4	0	0	0	0	0	0
	Liver	6	1	2	2	2	1	0	0	0	0	0
	Pancreas	42	22	10	8	4	0	1	0	1	1	1
	Ribcage	1	1	1	1	1	1	1	1	1	1	1
	Small Bowel	17	12	7	7	6	2	2	1	1	0	0
	Spleen	5	4	1	1	1	0	0	0	0	0	0
	Stomach	2	1	0	0	0	0	0	0	0	0	0
	UB	15	1	0	0	0	0	0	0	0	0	0
Dice equal to zero	AG	75	62	13	9	6	4	0	2	2	1	1
	Brain	28	42	18	13	15	10	13	18	12	5	5
	Colon	7	4	1	1	0	1	0	2	0	0	0
	Eyeballs	83	71	52	37	34	13	6	4	3	1	1
	GB	142	109	70	44	45	24	20	19	11	9	4
	Heart	2	2	1	0	0	0	0	0	0	0	0
	Kidneys	36	20	6	4	4	0	0	0	0	0	0
	Liver	5	1	1	2	2	1	0	0	0	0	0
	Pancreas	60	26	11	10	4	1	2	1	1	1	1
	Ribcage	1	2	1	1	1	1	1	1	1	1	1
	Small Bowel	19	12	6	6	6	2	2	1	0	0	0
	Spleen	12	8	4	3	2	0	1	0	0	0	0
	Stomach	6	2	1	1	1	0	0	0	0	1	0
	UB	20	1	1	0	0	0	0	0	0	0	0
Dice < 0.5	AG	94	76	25	14	8	5	1	4	2	1	1
	Brain	32	42	19	13	17	12	20	22	16	7	8
	Colon	11	6	1	2	1	1	0	2	0	0	0
	Eyeballs	83	78	54	41	39	15	8	6	4	4	1
	GB	164	130	82	52	55	27	25	19	18	10	4
	Heart	6	2	1	0	0	0	0	0	0	0	0
	Kidneys	60	27	15	8	7	0	1	0	0	0	0
	Liver	8	3	2	2	2	2	1	1	0	1	0

**Pancreas**	88	39	18	13	8	1	2	1	1	1	1
**Ribcage**	1	2	1	1	1	1	1	1	1	1	1
**Small Bowel**	25	18	9	10	10	2	2	1	1	0	0
**Spleen**	28	12	5	4	3	3	2	2	1	1	1
**Stomach**	12	5	1	1	1	0	0	1	1	1	1
**UB**	25	1	1	0	1	1	0	0	0	0	0

Supplementary figures 1 and 2 show the p values of spearman correlation for different organs included in tasks #1 and task #2, respectively.

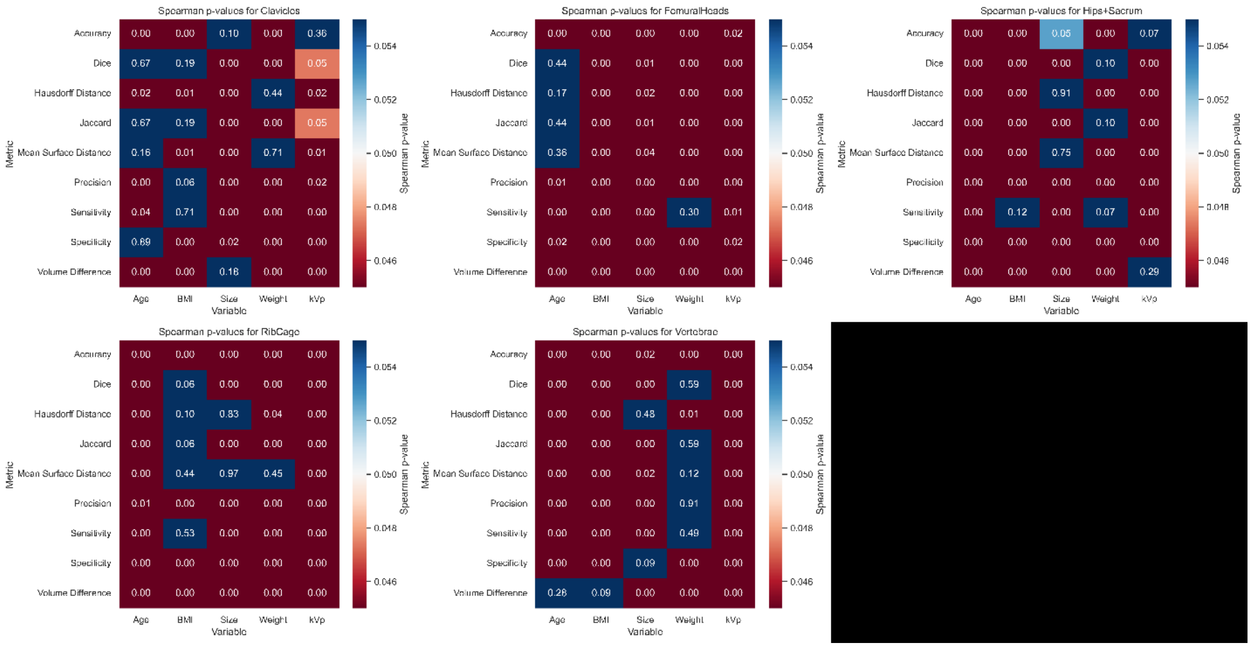

Supplementary figure 1. P-values for task #1 spearman correlation test.

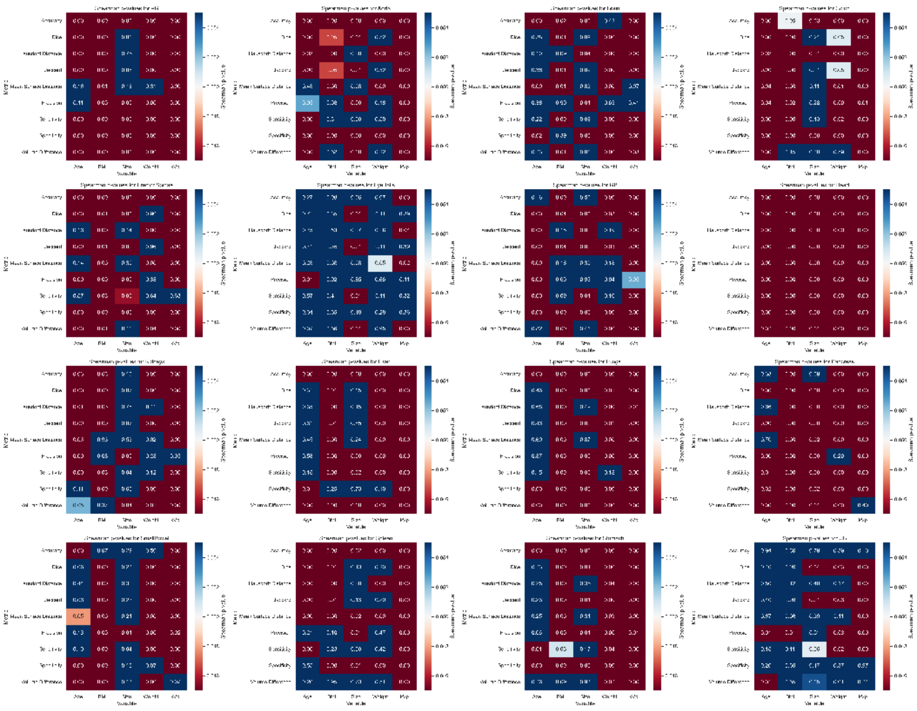

Supplementary figure 2. P-values or task #2 spearman correlation test.

Supplementary figure 3 presents the number of images with faulty segmentation on th low dose CT images.

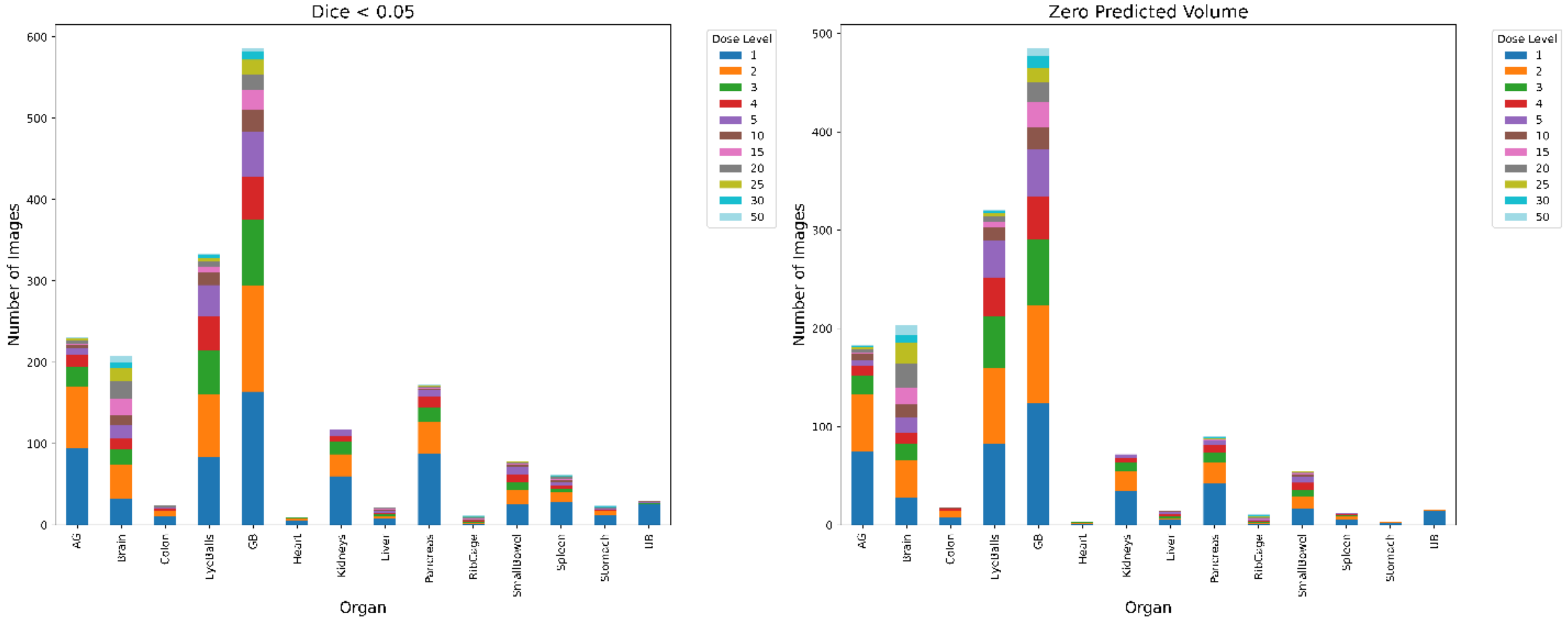

Supplementary figure 3. The number of outliers categorized by two criteria: Dice values less than 0.05 (left) and empty segmentation outputs (right) generated by FD-nnU-Net models. The legend shows the dose levels in percent.

Supplementary Figure 4 shows the axial images comparing the performance of FD-nnU-Net versus LD-nnU-Net models.

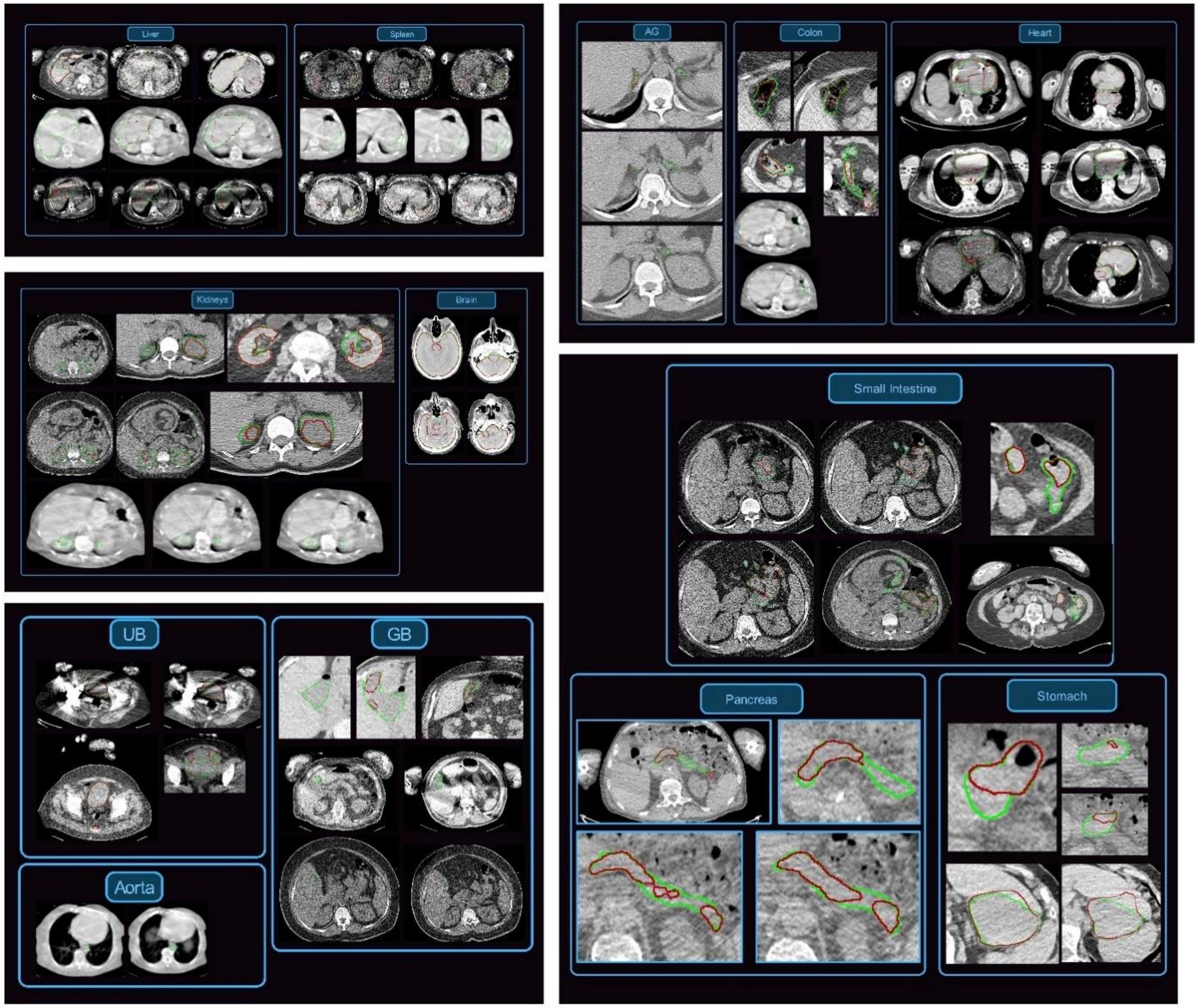

Supllementary Figure 4. Representative axial slices showing images comparing the segmetnation masks delineated by two of our LD-nnU-Net and FD-nnU-Net models demonstrating the robust and excellent performance of our LD models when the FD models failed to delineate the organs correctly. The green contour shows the LD-nnU-Net whereas the red contour shows the FD-nnU-Net segmentation output.

Supplementary figures of 5, 6, 7, and 8 represents example of datasets of #1, #2, #5, and #5 respectively.

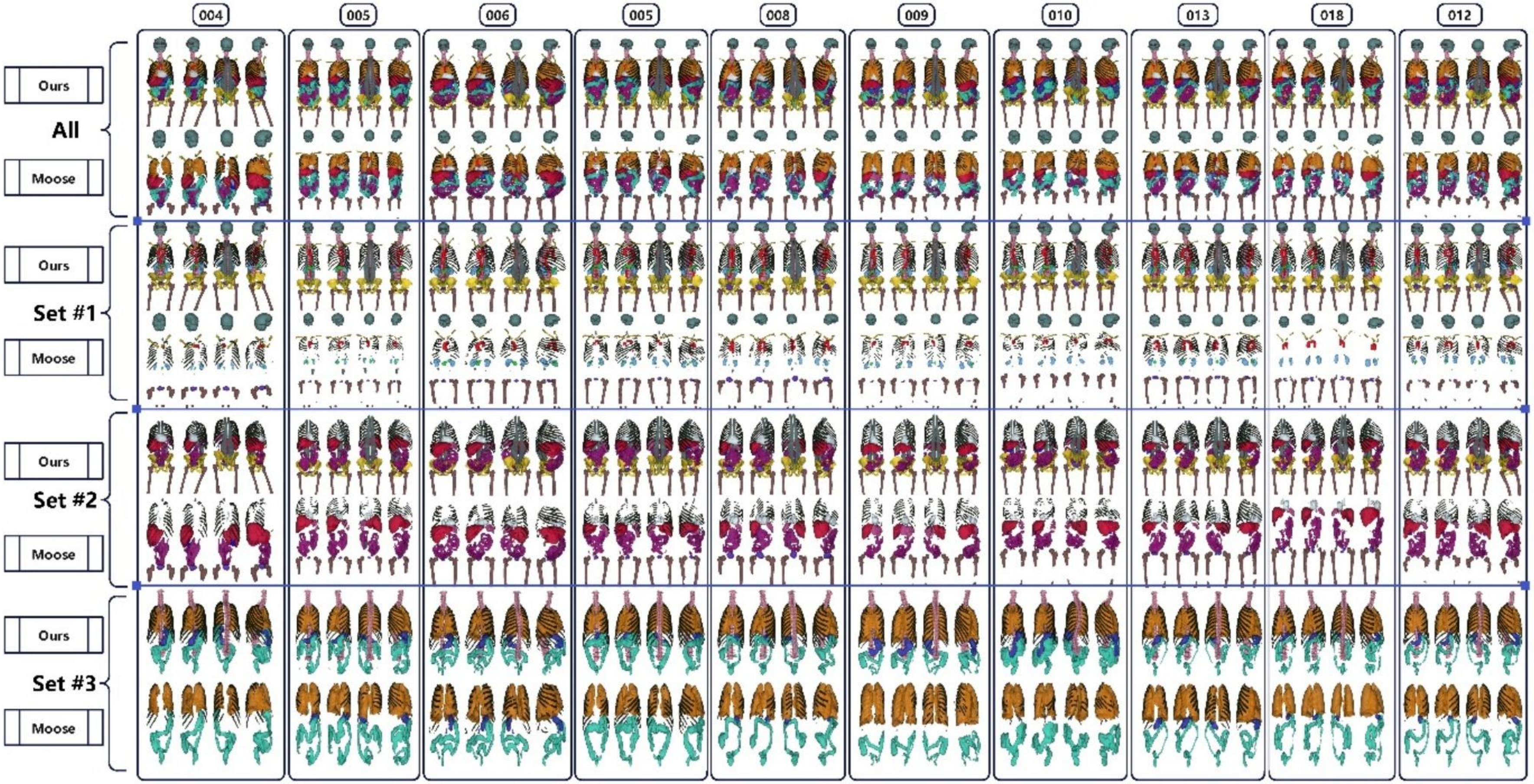

Supplementary figure 5. Comparison of our models vs MOOSE model on 10 images included in dataset #1. Color guide: AG: Blue, Aorta: Bright Red, Brain: Teal, Clavicle: Golden Yellow, Colon: Aqua Green, Eyeballs: Burgundy, FH: Muted Red, GB: Bright Blue, Sacrum: Mustard Yellow, Hips: Bright Yellow, Kidneys: Sky Blue, Liver: Crimson Red, Lungs: Orange, Pancreas: Emerald Green, Erectus Spinae: Dark Teal, Ribs: Olive Green, Small Bowel: Magenta, Spleen: Fuchsia, Stomach: Royal Blue, UB: Violet, Vertebrae: Light Pink, Heart: Pale Blue.

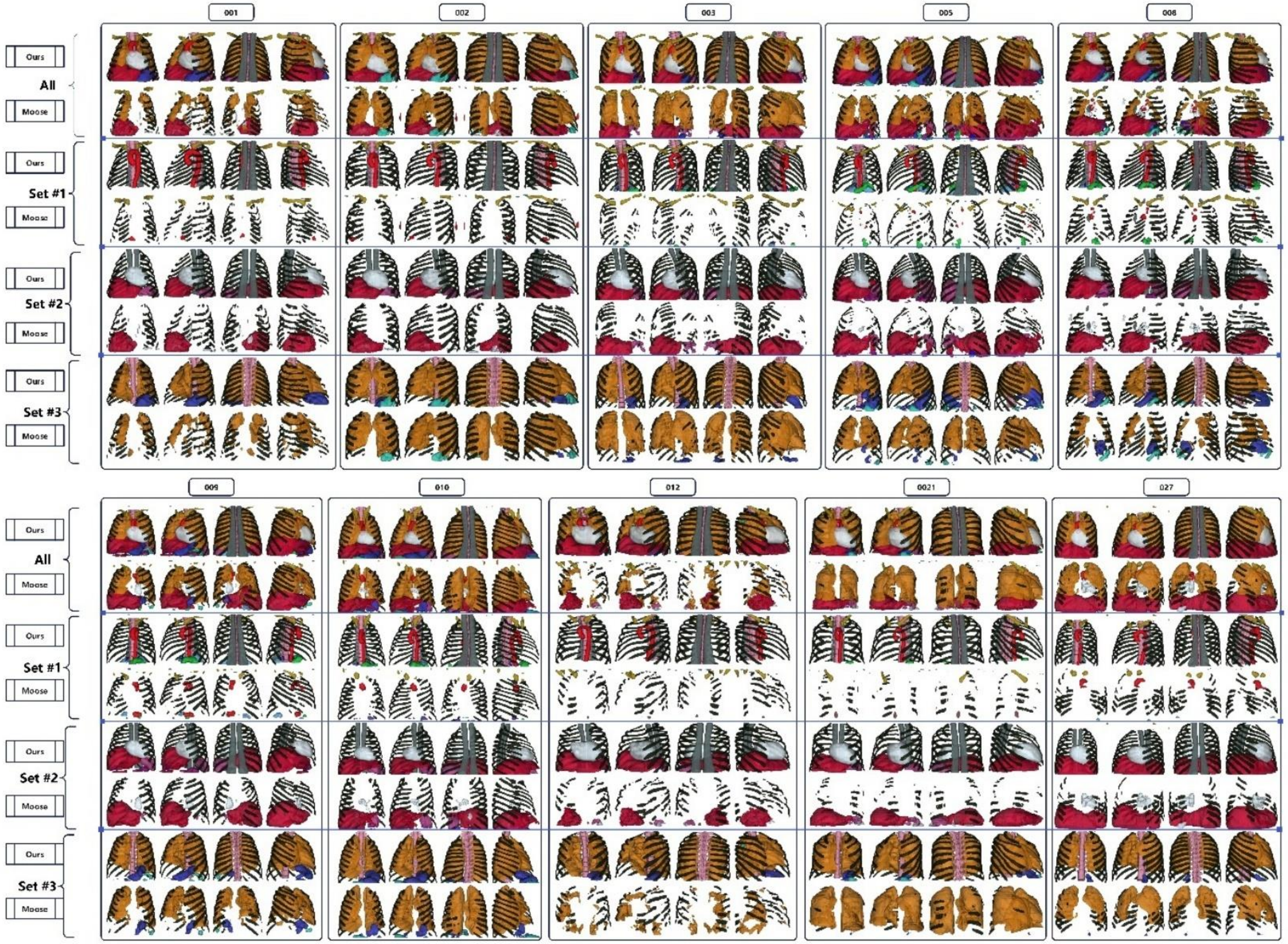

Supplementary figure 6. Comparison of our models vs MOOSE model on 10 images included in dataset #2. Color guide: AG: Blue, Aorta: Bright Red, Brain: Teal, Clavicle: Golden Yellow, Colon: Aqua Green, Eyeballs: Burgundy, FH: Muted Red, GB: Bright Blue, Sacrum: Mustard Yellow, Hips: Bright Yellow, Kidneys: Sky Blue, Liver: Crimson Red, Lungs: Orange, Pancreas: Emerald Green, Erectus Spinae: Dark Teal, Ribs: Olive Green, Small Bowel: Magenta, Spleen: Fuchsia, Stomach: Royal Blue, UB: Violet, Vertebrae: Light Pink, Heart: Pale Blue.

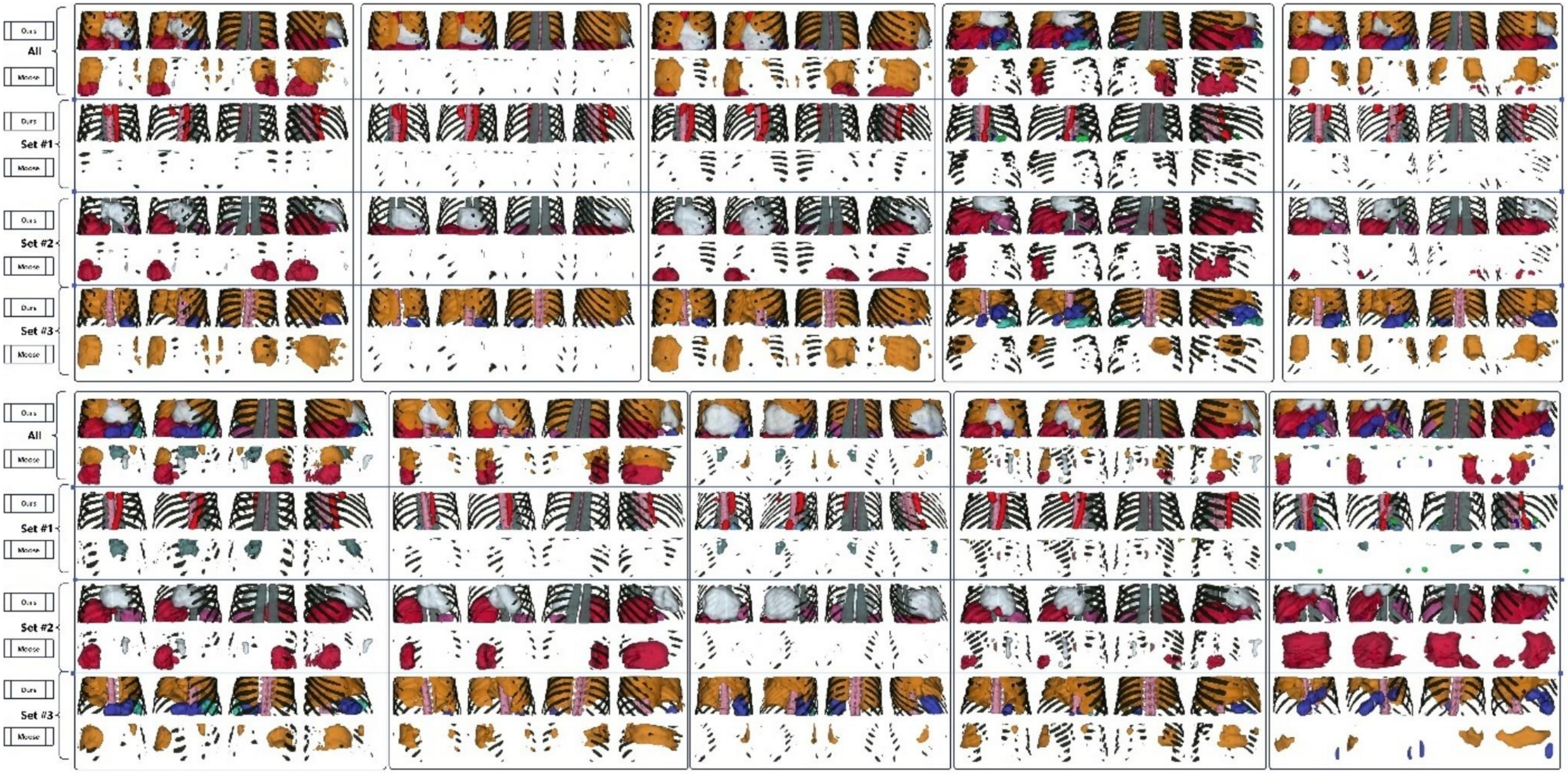

Supplementary figure 7. Comparison of our models vs MOOSE model on 10 images included in dataset #3. Color guide: AG: Blue, Aorta: Bright Red, Brain: Teal, Clavicle: Golden Yellow, Colon: Aqua Green, Eyeballs: Burgundy, FH: Muted Red, GB: Bright Blue, Sacrum: Mustard Yellow, Hips: Bright Yellow, Kidneys: Sky Blue, Liver: Crimson Red, Lungs: Orange, Pancreas: Emerald Green, Erectus Spinae: Dark Teal, Ribs: Olive Green, Small Bowel: Magenta, Spleen: Fuchsia, Stomach: Royal Blue, UB: Violet, Vertebrae: Light Pink, Heart: Pale Blue.

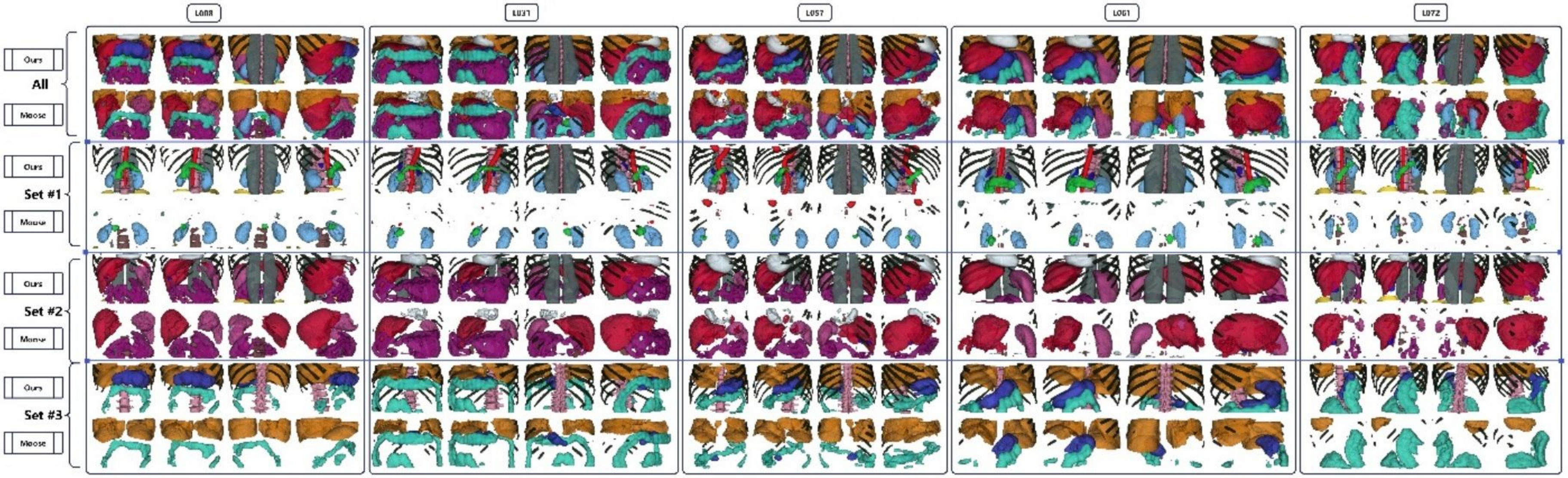

Supplementary figure 8. Comparison of our models vs MOOSE model on 5 images included in dataset #5. Color guide: AG: Blue, Aorta: Bright Red, Brain: Teal, Clavicle: Golden Yellow, Colon: Aqua Green, Eyeballs: Burgundy, FH: Muted Red, GB: Bright Blue, Sacrum: Mustard Yellow, Hips: Bright Yellow, Kidneys: Sky Blue, Liver: Crimson Red, Lungs: Orange, Pancreas: Emerald Green, Erectus Spinae: Dark Teal, Ribs: Olive Green, Small Bowel: Magenta, Spleen: Fuchsia, Stomach: Royal Blue, UB: Violet, Vertebrae: Light Pink, Heart: Pale Blue.

Supplementary figure 9 shows an example of a challenging case for analytic body contour segmentation with an excellent output generated by task #3 LD-nnU-Net model.

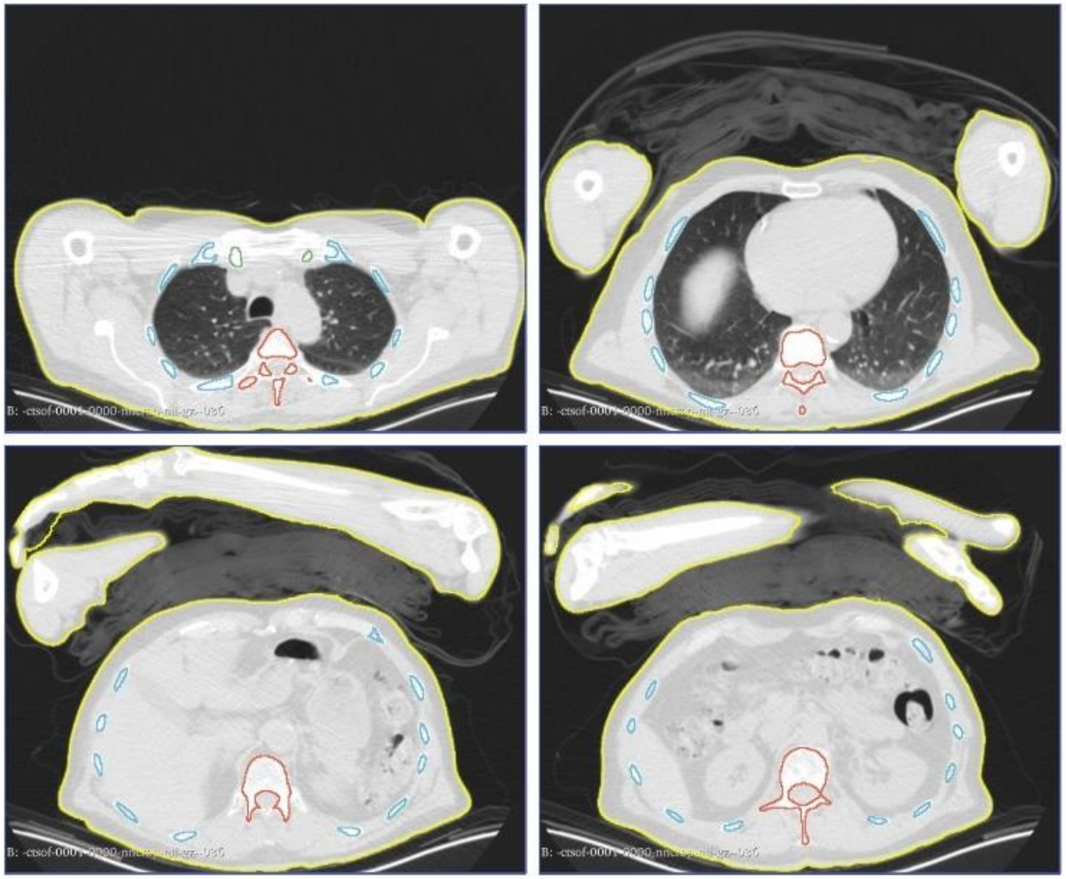

Supplementary figure 9. A challenging case segmented in task #3.
